# An agent-based model of metabolic signaling oscillations in *Bacillus subtilis* biofilms

**DOI:** 10.1371/journal.pcbi.1013746

**Published:** 2025-12-04

**Authors:** Obadiah J. Mulder, Maya Peters Kostman, Abdulrahmen Almodaimegh, Michael D. Edge, Joseph W. Larkin

**Affiliations:** 1 Department of Quantitative and Computational Biology, University of Southern California, Los Angeles, California, United States of America; 2 Departments of Biology and Physics, Boston University, Boston, Massachusetts, United States of America; Pázmány Péter Catholic University: Pazmany Peter Katolikus Egyetem, HUNGARY

## Abstract

Microbes of nearly every species can form biofilms, communities of cells bound together by a self-produced matrix. It is not understood how variation at the cellular level impacts putatively beneficial, colony-level behaviors, such as cell-to-cell signaling. Here we investigate this problem with an agent-based computational model of metabolically driven electrochemical signaling in *Bacillus subtilis* biofilms. In this process, glutamate-starved interior cells release potassium, triggering a depolarizing wave that spreads to exterior cells and limits their glutamate uptake. More nutrients diffuse to the interior, temporarily reducing glutamate stress and leading to oscillations. In our model, each cell has a membrane potential coupled to metabolism. As a simulated biofilm grows, collective membrane potential oscillations arise spontaneously as cells deplete nutrients and trigger potassium release, reproducing experimental observations. We further validate our model by comparing spatial signaling patterns and cellular signaling rates with those observed experimentally. By oscillating external glutamate and potassium, we find that biofilms synchronize to external potassium, providing a potential mechanism for previously observed oscillation synchronization. By tracking cellular glutamate concentrations, we find that oscillations evenly distribute nutrients in space: non-oscillating biofilms have an external layer of well-fed cells surrounding a starved core, whereas oscillating biofilms exhibit a relatively uniform distribution of glutamate. Our work shows the potential of agent-based models to connect cellular properties to collective phenomena and facilitates studies of how inheritance of cellular traits can affect the evolution of group behaviors.

## Author summary

Bacteria frequently form communities of cells called biofilms. These are large conglomerates of microbes that use self-produced polymers to bind together. Biofilms are very dense and nutrient diffusion within them is slow. If a biofilm is grown in a low-nutrient environment, it can eventually reach a size at which nutrients cannot diffuse to the center of the biofilm before being absorbed by exterior cells. The species *Bacillus subtilis* can respond to this condition with collective electrochemical signals. Initially, interior cells become metabolically stressed and signal by releasing potassium ions. This has a twofold effect: first, signaling enhances the ability of the signaling cell to absorb nutrients, and second, it slows nutrient uptake for surrounding cells. When enough cells become stressed and signal, it slows nutrient absorption across the biofilm and triggers a wave of signaling. This wave stalls growth, allowing nutrients to diffuse to interior cells. Once signaling stops and nutrients are absorbed, cells become stressed and signal again. This produces oscillating signals and cyclic growth. Several previous models of this system exist, either modeling only local interactions or assuming some level of homogeneity to model global dynamics. Here, we use an agent-based model overlaid on a simplified model of nutrient diffusion to simulate an entire biofilm. With this added flexibility, we are able to make predictions for the behavior of nearby biofilms and confirm that signaling results in a more equal distribution of glutamate across the biofilm.

## Introduction

Bacterial biofilms are large communities of cells that exist in nearly every environment [1]. They are bound together by an extracellular matrix that provides both stability and protection [2–4]. Biofilms exhibit a variety of emergent behaviors that give biofilm-dwelling microbes advantages unavailable to planktonic cells [5–7]. For example, cells within biofilms differentiate into heterogeneous phenotypes [8–11], divide labor [12,13], and coordinate behavior via chemical signals [14–17]. These group phenomena have led some researchers to assert that biofilms represent a transition between single-celled and multicellular life [18,19].

A striking multicellular behavior is the presence of cell-to-cell electrochemical signals that influence metabolism in *Bacillus subtilis* biofilms [20,21]. As a biofilm expands, fewer nutrients penetrate to the center; most are consumed by exterior cells [22,23]. The paucity of nutrients in the interior raises a problem: if interior cells are starved, the integrity of the biofilm is at risk [20]. *In vitro B. subtilis* biofilms exhibit a behavior that seems to allow them to navigate this challenge. When interior cells become starved, they release potassium, which depolarizes nearby cells and hampers their ability to absorb glutamate. In turn, nearby cells become distressed, release potassium, and hyperpolarize, eventually leading to a wave of potassium release. This wave propagates to the biofilm exterior [21]. It has been hypothesized that glutamate consumption among cells in the exterior slows down enough that glutamate can diffuse to the center [20]. Once interior cells have enough glutamate, they cease releasing potassium, allowing exterior cells to repolarize and resume consumption, eventually leading to stress and another wave of potassium release.

These repeated waves of potassium release have been referred to as a form of microbial “signaling” [21,24,25]. Potassium signaling has been proposed to efficiently allocate nutrients at the colony level [20,26] and is heterogeneous at the cellular level. Some cells participate in signaling and hyperpolarize during signaling waves, whereas others do not [27]. It is unknown how cellular variation in signaling behavior affects biofilm-level properties, such as distributions of nutrients. In order to answer this question, we need models that can connect cell-level properties, such as signaling state and inheritance of signaling behavior, to colony-level phenomena.

Several computational models of *B. subtilis* signaling behavior have been introduced to explore hypotheses about the causes and effects of signaling. Zhai and colleagues [28] proposed an agent-based model to explain observations they had made about signaling. Their *in vitro* experiments revealed that a roughly constant proportion of cells signal each oscillation, that the same cells tend to release potassium in repeated signaling waves, and that signaling behavior is weakly heritable—that is, daughter cells of signaling cells are more likely than average to participate in signaling waves. They modeled signaling as a percolation process in which a cell only signals during a depolarization wave if it both has a binary trait that predisposes it to signaling and is adjacent to another signaling cell in the biofilm. Using an agent-based model in which agents represent individual cells allowed them to test whether signaling in this manner would transmit a signal across the biofilm consistently. However, their model focused on small sub-regions of the biofilm to match the limitations of their experimental system—a roughly 35× 230 rectangle of cells at the edge of the biofilm, where the colony is close to two-dimensional. Their model also did not include nutrient diffusion or uptake, preventing its use for studying how individual cell behaviors affect the distribution of nutrients or growth of the biofilm.

Other models of *B. subtilis* depolarization waves are based on systems of differential equations. Martinez-Corral et al. [24] produced a model of a one-dimensional slice of the biofilm, extending from the center to an edge. Ford et al. [25] extended this to two dimensions, simulating a complete biofilm. Both models aimed to capture signaling and nutrient patterns at the scale of an entire biofilm. These models explicitly simulate nutrient diffusion and metabolism and feature signaling that operates through mechanisms that depend on internal glutamate concentration, providing powerful and accurate recreation of biofilm-wide signaling dynamics. However, modeling these complex interactions at a larger scale using differential equations comes at the cost of resolution. These models describe phenomena on the scale of the biofilm but do not distinguish individual cells. Their advantages are thus opposite those of the agent-based model of Zhai and colleagues, but neither can describe the effects of individual-cell behaviors on broad patterns of nutrient distribution or signaling.

The model we propose strikes a compromise between the flexibility and resolution of the agent-based approach of Zhai and colleagues [28] and the scalability of ODE models. Our approach is agent-based, but the agent-based elements are overlaid on a simplified version of the ODE model developed by Martinez-Corral et al. [29]. Via this hybrid strategy, our model retains some of the benefits of both previous approaches. Our model enables simulation at the scale of an entire flow-cell biofilm [30,31], comprising approximately 51,000 individual cells, each with unique potassium, glutamate, membrane potential, and signaling dynamics.

**Fig 1:**
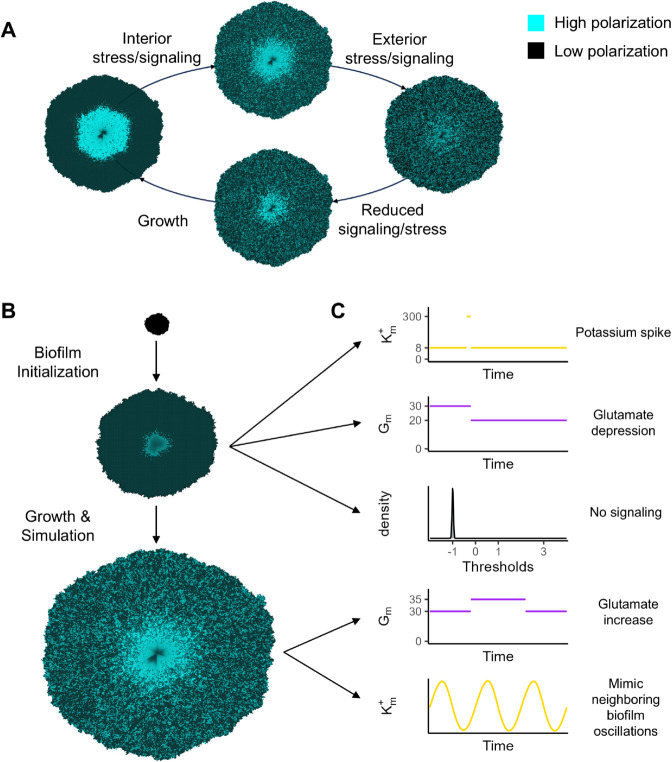
Model schematic. (A) shows the cycle of oscillations: growth causing interior stress, leading to signaling (indicated by cyan cells) and exterior stress, causing slowed growth and a reduction in stress, and finally back to resumed growth. (B) shows our simulation process, beginning with a very small cluster of cells, growing it for a period of time without simulating nutrients, and then growing to full size and running for many iterations with nutrient and signaling simulation. Oscillations arise naturally during this second phase. (C) shows the questions we pursue, including testing the effects of a neighboring biofilm oscillating and of suppressing signaling.

We validate our model by comparing the behaviors of simulated biofilms with those observed in experiments, including signaling patterns at local and colony-wide scales, response to various stressors, and growth patterns (Fig 1C). We show that many of the distinctive features of *B. subtilis* signaling, including waves of depolarization and the fraction, identity, and descent of cells that participate in signaling, can emerge naturally from our model. We then demonstrate the application of our model by exploring open questions regarding synchronization of oscillations among neighboring biofilms [26] and the effect of signaling on glutamate distribution.

## Results

### Model overview

Our model aims to describe an oscillating hyperpolarization-depolarization behavior observed in *B. subtilis* biofilms grown in flow cells [20,21]. In such scenarios, when a biofilm grows past a certain size, metabolically stressed interior cells release potassium ions. The primary source of nitrogen in flow-cell experiments is glutamate [20], and cells absorb glutamate via a transporter whose activity depends on membrane potential. This transporter is more efficient when the cell is hyperpolarized—that is, when there is a greater charge differential between the interior of the cell and the extracellular media [32]. By releasing charged potassium ions, stressed cells increase their membrane polarization and therefore their ability to absorb nutrients.

Releasing potassium ions has an additional effect of depolarizing nearby cells. Prindle et al. [21] hypothesized that when interior cells are extremely stressed and release a sufficiently large amount of potassium, they can depolarize surrounding cells enough to slow their nutrient uptake. If enough cells release potassium, a chain reaction can be triggered in which nearby cells become depolarized, undergo metabolic stress, and then release ions and hyperpolarize in response. Ion release can be thought of as a form of signaling, albeit one that has direct effects on cell physiology. If enough cells signal, it can lead neighboring cells to signal, causing a wave of depolarization to spread across the biofilm.

As the wave of depolarization crosses the biofilm, nutrient absorption across the entire colony slows. This eventually allows nutrients to diffuse to the interior of the biofilm and reduce metabolic stress. A side effect of reduced nutrient uptake is a corresponding reduction in growth [33], particularly in exterior cells where most biofilm expansion occurs [20,34]. After the wave of depolarization reaches the biofilm exterior and nutrient diffusion reduces interior stress, growth can resume. Consequently, whereas there is consistently rapid growth when the biofilm is small, once it surpasses a threshold size—determined by nutrient concentration in the media and the biofilm’s shape and density—it transitions to periodic growth, with growth pausing when the exterior of the biofilm is depolarized.

Our model describes the signaling waves that appear to drive these oscillations in growth (Fig 1A). We developed an agent-based model that explicitly simulates each cell spatially on a two-dimensional plane. Our model is hexagonal (to mimic the approximate 6-neighbor structure of a 2D biofilm [27]), and can be run at the scale of an entire flow-cell biofilm, with a radius of approximately 145 cells. We model glutamate as diffusing into the biofilm from outside and being consumed by cells; uptake of glutamate causes a cell’s internal glutamate level to increase. When cells are below an individual-specific threshold level of internal glutamate, they release potassium ions, allowing faster glutamate uptake.

Intracellular glutamate (*G*_*i*_) and potassium (*K*_*i*_), extracellular glutamate (*G*_*e*_) and potassium (*K*_*e*_), and cell membrane potential (*V*) are regulated by four equations taken from Martinez-Corral et al. [29] with simplifications (Eq S1, Eqs S3-S5, see S2 Tab for parameter values; see S1 Text Sect 3.4 for a discussion of parameters, including correction of a Nernst potential prefactor from previous models of *B. subtilis* signaling). Each cell has a signaling threshold ${𝒯}_{i}$—when a cell’s internal glutamate drops below ${𝒯}_{i}$, the cell signals. ${𝒯}_{i}$ is treated as a property of the cell that remains fixed throughout the cell’s lifespan. Cells pass their signaling threshold to their offspring, with a certain amount of noise, causing signaling behavior to be partially heritable, as observed *in vitro* [28]. (We use the term “heritable” to refer to the correlation between mother and daughter cells, without assuming that the source of variation between lineages is genetic, which is unlikely in clonal biofilms.) An illustration of the potassium, glutamate, and membrane potential for a single cell during a signaling wave is shown in S1 Fig.

Although the equations governing biofilm behavior are based on those of Martinez-Corral et al. [29], we made modifications for use in an agent-based model. For computational tractability, we discretized coarsely with respect to time, applying the equations every time step (“tick”). A tick represents a period of approximately one minute, an interval with respect to which potassium diffuses rapidly (S1 Text Sect 3.4.1, [29]). This coarse time grid allowed us to model potassium diffusion simply by averaging it across the biofilm each tick. Glutamate diffuses more slowly than potassium [21,35], so we model its diffusion, albeit in a simplified way (described in S1 Text Sect 3.1, see S1 Text Sect 3.6 for validation). Each tick, basal glutamate in the media (*G*_*m*_) diffuses into the biofilm and is absorbed by cells according to Eq S1.

We initialized biofilms with a small number of cells such that glutamate diffused to the center easily. We then allowed them to grow to a radius of approximately 145 cells (a population of approximately 51,000), at which point we stopped growth (Fig 1B). At each tick during the growth phase, we selected one-fortieth of the cells on the perimeter of the biofilm network, uniformly at random and with replacement, to reproduce. This produced growth consistent with the doubling time of *B. subtilis* (between 45 minutes and 6 hours [36–38]). Each daughter cell was placed in one of the empty nodes adjacent to the parent. Its signaling threshold was drawn from a truncated normal distribution with mean equal to the parent’s threshold, *σ* (corresponding to the standard deviation of a non-truncated normal distribution) of 1, and bounds of [0, 3] (further described in S1 Text Sect 2).

Signaling arose naturally during biofilm growth, and by the end of the growth phase (once the biofilm had reached a population of approximately 51,000 cells) signaling oscillations were stably occurring. If growth were to be allowed much past this population size, oscillations would eventually collapse as the biofilm becomes too large and the center cells starve. Thus, to study oscillation dynamics while avoiding collapse, we continued simulating without growth for a total of 3000 ticks. This simulated period corresponds to approximately 48 hours, longer than *in vitro* biofilms have been observed to maintain oscillatory behavior. To replicate previous studies and make new predictions, we simulated biofilms under a variety of conditions, including reduced and increased basal glutamate, oscillated basal glutamate and potassium, and a short flood of potassium to depolarize the biofilm (Fig 1C).

### Model validation

#### Patterns of signaling.

We initially explored our model by replicating behaviors and findings from previous work. We first examined whether our model produced simulated biofilms in which signaling oscillations behave similarly to *in vitro* observations. At a gross level, videos of oscillations in *in vitro* biofilms and in our simulated biofilms reveal many similar features (S1 Vid and S2 Vid).

**Fig 2:**
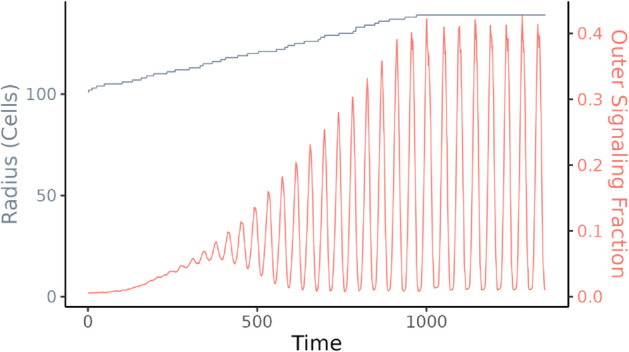
Oscillations increase in magnitude with biofilm size. Radius of the biofilm (gray) and fraction of signaling cells (red) in the outer region of a simulated growing biofilm. The radius indicates the distance of the cell farthest from the center. Growth is limited to a radius of approximately 145 cells. If allowed to grow larger, signaling will eventually collapse.

Martinez-Corral et al. [24] observed that oscillations generally begin at a radius of 200-350 μm under environmental conditions identical to those in our model (30 mM glutamate). We found oscillations to start at a radius of around 110 cells (Fig 2), which corresponds to approximately 220-330 μm [39,40].

Zhai et al. [28] found that near the edge of the biofilm, approximately 43% of cells signal during the peak of each oscillation. This observation motivated their investigation of signaling in terms of percolation theory—43% is near the minimum fraction of signaling cells that guarantees a signal moving between adjacent cells can cross the biofilm, given their other assumptions. Our simulated biofilms behave similarly, with approximately 42% of cells in the outer layers of the biofilm signaling at the height of each signaling oscillation (Fig 2).

**Fig 3:**
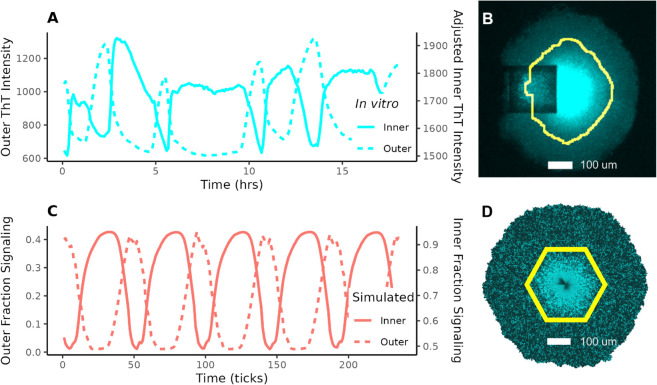
Comparison of signaling patterns between an *in vitro* and a simulated biofilm. (A) and (C) show oscillations in the interior and exterior of the biofilm. (A) is an *in vitro* observation: time is given in hours and the y-axis shows the average Thioflavin-T (ThT)^†^ intensity in each region. Note that the interior has much higher ThT intensity than the exterior. (C) is a simulated version of the same. (B) is an *in vitro* fluorescence image of a signaling biofilm (cyan represents ThT intensity; the square is a cell loading trap) and (D) is snapshot from our model, both with the boundary between inner and outer cells highlighted (yellow). ^†^ThT is a stain used to detect membrane polarization; polarized cells absorb it and exhibit fluorescence [21,41].

In experimental time-lapse images of biofilm signaling, the interior and exterior of the biofilm oscillate approximately in antiphase, with the interior exhibiting much higher polarization (S1 Vid, Fig 3A). *In vitro*, the division between the interior and exterior (defined by oscillation) appears sharp (Fig 3B). In our simulations, we observed the same boundary (S2 Vid, Fig 3D). The difference in polarization can also be observed by comparing the vertical axes for inner and outer cells in Fig 3(A) and 3(C).

A discrepancy between our simulated biofilm (Fig 3D) and the *in vitro* image (B) is the dark core of cells at the center of (D). They have some glutamate—more than they would if signaling were not present (S2 Fig)—but not enough to signal and absorb ThT. Liu et al. [20] observed cell death at the center of signaling biofilms. This has been further confirmed by our own observations of *in vitro* experiments. Because ThT staining is a property of individual cells in our model and there is no diffusion, cell movement, or death, these starved cells show up more clearly in our simulations than they do *in vitro*.

Throughout this paper, we will refer to measurements for the exterior and interior of the biofilm. The exterior is defined as the region where glutamate would diffuse if signaling were not present, and the interior is defined as everything more than 8 cells toward the center from that depth. The 8-cells-wide buffer region ensures that we capture the separate dynamics in the two regions. When defined this way, the bound falls very close to the point at which signaling oscillations change phase (S1 Text Sect 1.2, S3 Fig).

#### Single-cell signaling behavior.

Larkin et al. [27] found a bimodal distribution of cell-level membrane potentials (as represented by ThT stain intensity) during signaling peaks. Cells that had recently signaled had substantially more negative membrane potentials than those that had not. We found ThT to also have a bimodal distribution in our simulations and used this to define signaling vs. non-signaling cells. Cells with higher ThT (corresponding to hyperpolarization) were classified as signalers, whereas those at the lower mode were classified as nonsignalers (S4 Fig).

At the individual-cell level, signaling behavior is consistent across oscillations: cells that signal in a given wave are more likely to participate in other waves of signaling. To characterize this consistency, we used *in vitro* lineage tracing across two oscillations, again focusing only on exterior cells. We found that across a pair of oscillations, 38% of cells signaled in both waves (compared with approximately 18% expected if signaling participation is independent between waves), 50% did not participate in either signaling wave, and 12% switched their signaling behavior between waves (with roughly half going either direction). These proportions are inconsistent with the null hypothesis that cell-level signaling behavior is independent between waves (Fisher’s exact test *p* < 10^−24^). We then measured pairwise consistency in our simulations to compare with our *in vitro* findings. In our simulations, we observed similar behavior, with 41% consistently signaling, 57% consistently not signaling, and just 2% switching (Table 1).

**Table 1:** A comparison between individual-cell behaviors observed *in vitro* and those predicted by our simulations.

	Observed	Simulated
Signaling Fraction	0.43 ± 0.02	0.42 ± 0.001
Signaler Recurrence	0.60 ± 0.1	0.63 ± 0.003
Non-signaler Recurrence	0.78 ± 0.1	0.73 ± 0.002
Consistent Signaling Fraction	0.38 ± 0.03	0.41 ± 0.002
Consistent Non-signaling Fraction	0.50 ± 0.03	0.57 ± 0.003
Inconsistent Fraction	0.12 ± 0.02	0.02 ± 0.001

All simulated values are for exterior cells only. Signaling fraction and recurrence rates are from Zhai et al. [28]. Signaling fraction is the maximum proportion of cells simultaneously signaling during each oscillation. The recurrence rates are the probabilities that a daughter cell will exhibit the same signaling state as its parent in a given oscillation. Errors for all results are standard errors. Zhai and colleagues did not give error rates for their calculations, so these are estimates. Consistency fractions are based on data from Larkin et al. [27], with errors estimated as for a binomially distributed observation. S1 Tab is an extended version of this table with data from inner cells and the total population, additional measures, and a description of the standard error estimation.

In our simulations, we also examined consistency across many waves of signaling and across an entire signaling oscillation, not just looking at a snapshot of signaling during the peak. Approximately 250,000 cells were tracked across 30 oscillations, and we recorded the fraction of oscillations during which they signaled. We found that 47% of cells consistently signaled (>90% of the time), 51% consistently did not signal (<10% of the time), and 2% were inconsistent. Note that this adds up to more than the mean of 43% signalers observed at oscillation peaks. This is due to the fact that more than 43% of cells signal each oscillation, but some signal before and some after each peak. This is also why the number of consistent nonsignalers decreased.

Finally, Zhai et al. [28] found that signaling behavior appears heritable—the daughter cells of cells that participate in signaling are more likely to participate in signaling themselves. In our model, the signaling thresholds of individual cells are noisily inherited, and this inheritance aligns with the observations of Zhai and colleagues. For example, with our selected values for signaling threshold inheritance, approximately 63% of daughter cells of signaling cells signal themselves, and approximately 73% of daughter cells of cells that do not participate in signaling also do not participate, close to the observations of Zhai et al. (Table 1). Further exploration of the effect of cell-level threshold on signaling appears in S1 Text Sect 2 and S5 Fig. To maintain comparability to the findings of Zhai and colleagues, we measured concordance of signaling status for each mother-daughter pair during a peak of signaling (though more measures are given in S1 Tab).

#### Responses to media perturbations.

**Fig 4:**
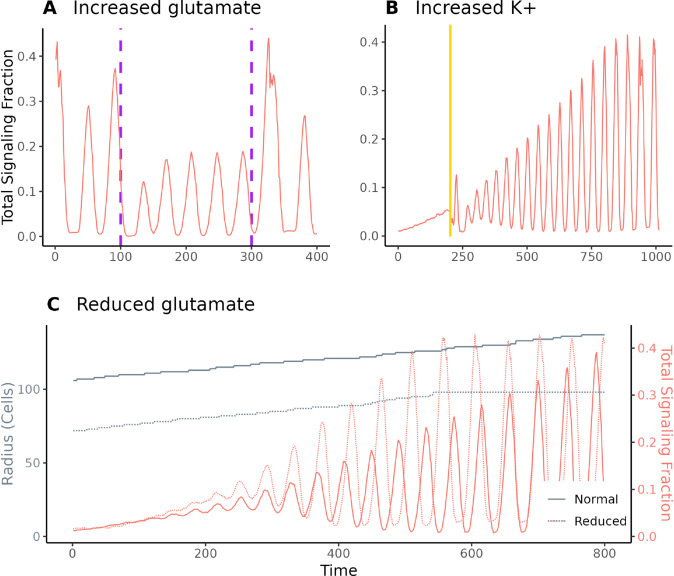
Effects of environmental conditions on signaling. (A) Increasing basal glutamate from 30 mM to 35 mM from ticks 100 to 300 in a biofilm that has been stably oscillating caused a depression in oscillation magnitude. (B) Depolarizing a growing biofilm by increasing basal potassium from 8 to 300 mM for five ticks (indicated by the gold band) caused a wave of signaling. This mimicked the methodology from Martinez-Corral et al. [29]. (C) By growing a biofilm in a reduced-glutamate environment (*G*_*m*_ = 20 mM) we caused oscillations to begin at a much smaller population size. Note that signaling rates in this figure are for the entire biofilm, not just outer cells, and are therefore sometimes higher than those reported elsewhere.

*B. subtilis* biofilm oscillation experiments have taken place within a strictly controlled environment, where glutamate, as the only nitrogen source in the media, acts as a limiting nutrient. Liu et al. [20] showed that, in such an environment, oscillations can decrease or stop in response to an increase in basal glutamate (the level of glutamate in the media surrounding the biofilm). Martinez-Corral et al. [24] further found that oscillations would begin at a smaller biofilm size if basal glutamate were reduced, and showed that depolarization during biofilm growth can cause a wave of signaling. Fig 4 shows the results of simulations intended to replicate these findings in our model. By increasing basal glutamate, we weakened oscillations (Fig 4A). By drastically increasing potassium to depolarize the biofilm, we caused an initial peak of signaling (Fig 4B), and by lowering basal glutamate, we triggered early oscillations (Fig 4C).

### Applications and predictions

#### Oscillation synchronization between adjacent biofilms.

In addition to reproducing previously observed experimental results, our model can make predictions that motivate new experiments. Liu et al. [26] found that two biofilms that are adjacent to each other will shift their oscillations to synchronize, but they did not identify a mechanism for this synchronization. We tested whether our model can replicate this synchronization via the oscillations in either potassium or glutamate outside of the biofilm. Initial testing showed that glutamate could trigger oscillations, but the magnitude of external glutamate oscillation required to synchronize signaling is more than would plausibly be caused by a neighboring biofilm (S6 Fig). We thus chose to focus on potassium as a potential trigger.

**Fig 5:**
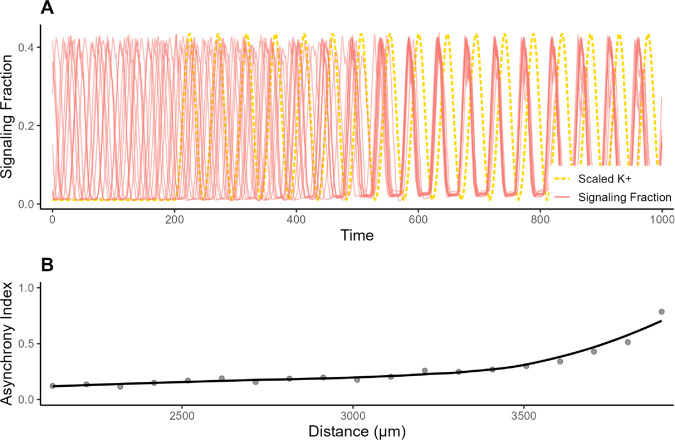
Effects of potassium oscillations on signaling synchronization over time. (A) A sample of 10 of 20 total simulated signaling trajectories. Each was simulated separately until stably oscillating, then basal potassium was oscillated as if another biofilm had begun weakly signaling 2000 μm away (tick 200). This linearly increased in magnitude for 400 ticks, until potassium oscillations reached the magnitude of a normally oscillating biofilm. Basal external potassium oscillations (gold) were the same for each simulation. (B) The above experiment was repeated at many different distances between the two biofilms. The Asynchrony Index is a measure of the range across the 20 simulations during each signaling trough between ticks 600 and 750, scaled to be between 0 and 1. Zero represents perfect synchrony (zero range), and one represents the degree of asynchrony observed with no potassium oscillation (see Methods). The black line is a smoothed trend line.

Potassium has an apparent mechanism by which it could regulate signaling in neighboring biofilms, since signaling involves the release of extracellular potassium, which can then diffuse through the media. We calculated the approximate diffusion of potassium between two biofilms in a flow cell, following the experimental design from Liu et al. [26]. For further details, see S1 Text Sect 3.5. When setting basal potassium to the level that would be caused by a neighboring oscillating biofilm and allowing our model to run as normal, we observed rapid synchronization. To illustrate this effect, we ran 20 simulations with the same basal potassium oscillations and calculated how closely they synchronized. After approximately 600 ticks, the horizontal variation across 20 replicates was 12% of what we would have expected without potassium oscillations (Fig 5). At a distance of about 4000 μm, the effect from oscillated potassium decreased to produce 80% of the expected variation.

#### Threshold effects.

**Fig 6:**
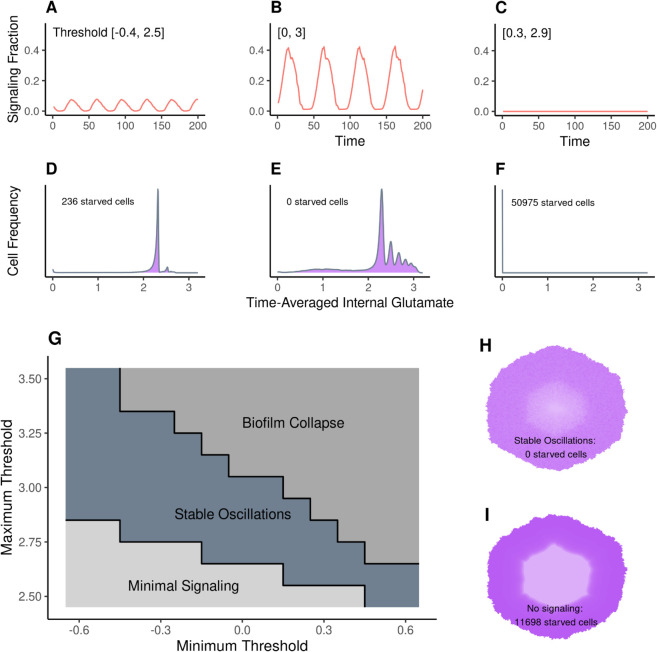
The effects of signaling threshold range on oscillation patterns and glutamate distribution. (A) shows the fraction of signalers over 200 ticks for a biofilm with low thresholds [−0.4, 2.5]. (Cells with signaling thresholds less than or equal to 0 never signal; more negative values of the lower bound lead to more cells that never signal.) (D) displays the corresponding internal glutamate levels averaged across time for all cells in the biofilm. 236 cells starved—had less than 10^−3^ mM internal glutamate on average after the end of biofilm growth. (B) and (E) display the same for a range of [0, 3], and (C) and (F) for [0.3, 2.9]. Note that (C) and (F) display a biofilm in which signaling behavior has collapsed. (G) shows a phase diagram of the region of maximum and minimum signaling thresholds in which we observe stable oscillations. The region of stable oscillations produces oscillations with a range of more than 20% between the lowest level of signalers and the highest (eg. (B)). Minimal signaling indicates a low average level of signaling (as seen in (A)), and the region of signaling collapse produces results like in (C). The trajectories for the simulations used to produce this phase plot are in S7 Fig. (H) is the time-averaged internal glutamate for the biofilm in (B), dark purple indicating higher internal glutamate. (I) is the same, except for a biofilm with no signaling, leading to the interior 11,698 cells starving. Versions of (H) for the other two boundary conditions can be found in S2 Fig.

In our model, the propensity of a cell to signal is determined by its signaling threshold. If a cell’s internal glutamate falls below its signaling threshold, then the cell will signal. The results described above were simulated using thresholds distributed over a truncated normal distribution, with a mean on the parental-cell signaling threshold, lower bound of 0, upper bound of 3, and *σ* of 1. To explore the effects of this distribution, we tested the signaling patterns and internal glutamate of biofilms across a variety of threshold bounds. We found that the distribution bounds must fall within a certain range in order for signaling to remain stable (Fig 6G, S7 Fig). If the maximum bound is too low, then signaling occurs, but only at very low levels (Fig 6A and 6D). There are never enough signalers to starve the exterior and trigger a wave of signaling, so only the interior cells signal. If the minimum bound is too high, then signaling collapses (Fig 6C and 6F). Too many cells signal simultaneously, and extracellular potassium increases to implausible levels. *In vitro*, we might expect chaotic, uncoordinated signaling and starvation of interior cells. Between these regimes, the biofilm exhibits stable oscillations (Fig 6B and 6E).

It has been proposed that potassium signaling promotes an even distribution of glutamate across the biofilm, plausibly improving the survival rate of interior cells [20,21]. We tested this idea by tracking the distribution of glutamate across cells in simulations that either did or did not include signaling behavior. By comparing the mean internal glutamate of cells across oscillations, we can see the effect of signaling. Without any signaling, exterior cells obtained substantial glutamate, but interior cells did not, with more than 11,000 (approximately 20% of all cells) reaching zero glutamate (Fig 6I). However, in simulated biofilms that signal, glutamate is much more evenly distributed across the biofilm, with no cells having zero glutamate (Fig 6H). Interestingly, the total amount of glutamate absorbed per cell decreases slightly when signaling is present (all available glutamate is absorbed without signaling, but with it there are times when depolarization is high and some glutamate is not absorbed), but the number of starved cells is much lower.

Even weak signaling produced substantially fewer starved cells than no signaling (Fig 6D–6E, S2 Fig), but only stable oscillations resulted in no starved cells. When signaling thresholds were too high, and thus signaling was triggered very easily, signaling collapsed. In this case, our model predicts that the vast majority of cells—including exterior cells—would starve due to potassium build-up causing complete depolarization and stopping all cells from absorbing nutrients (Fig 6F). These findings suggest that potassium signaling does promote even distribution of glutamate by slowing growth and allowing glutamate to diffuse to interior cells, potentially increasing the stability of the biofilm during periods of high metabolic stress.

## Discussion

We introduced a computational model of metabolic signaling in *B. subtilis* biofilms. Previous models of this behavior have either been limited in scope, focusing on local cellular behaviors and omitting nutrients, or broad in scope, but unable to capture heterogeneity in cell-level behavior. [25,28]. We have developed a model that bridges this gap, allowing the examination of the effect of cell-level behaviors on broader signaling patterns and the concentration of nutrients across the biofilm. We were able to replicate both individual-cell and biofilm-scale observations from previous work and new experiments, including oscillation and growth patterns, signaling in interior and exterior cells, and synchronization between neighboring biofilms. We also found support for the hypothesis that signaling results in a more even distribution of glutamate, which may extend the lifespan of a biofilm during periods of stress.

Prior models of *B. subtilis* signaling have adopted various assumptions about the effects of signaling on individual cells and the biofilm. On one hand, the models of Prindle et al. [21], Martinez-Corral et al. [24,29], and Ford et al. [25] encoded assumptions that imply that signaling will increase glutamate uptake for the signaling cell both by directly increasing the cell’s ability to absorb glutamate, and by suppressing glutamate absorption for neighboring cells.

On the other hand, the models in Larkin et al. [27] and Zhai et al. [28] prioritized the observation that hyperpolarized cells experience slower growth [26]. Larkin and colleagues hypothesized signaling to be costly to the individual cell but beneficial to the biofilm as a whole, as it promotes a more even distribution of glutamate. Further, they noticed that the fraction of cells that signal in a given wave was close to the minimum number of cells necessary for the signaling wave to propagate across the exterior of the biofilm as predicted by percolation theory [42] (where signalers are randomly distributed among non-signalers and a signal is propagated by direct contact between two signaling cells). They interpreted this observation as being consistent with the idea that signaling cells act altruistically, sacrificing their own growth to promote the integrity of the biofilm. Recent work by Han and Payne [43] has suggested that the slow growth of hyperpolarized cells may be an artifact of ThT staining inhibiting growth, raising questions about the degree of cost incurred by signaling cells. (However, the observations of Prindle et al. [21], who tested several stains before deciding on ThT, suggest that oscillatory signaling behavior itself is not dependent on ThT.)

Our model adopts assumptions similar to those of Prindle et al. [21] and Martinez-Corral et al. [29] that lead to signaling typically increasing the glutamate uptake of the signaling cell. At the same time, we replicate the heterogeneity in signaling behavior, the fraction of signaling cells, and the individual-level consistency of signaling across waves emphasized by Larkin et al. [27] and Zhai et al. [28]. Thus, the individual-cell-level signaling patterns observed by the latter studies—and particularly a fraction of signaling cells near the percolation-theory threshold for signal transmission—can be attained without an explicit trade-off between individual-level growth and group-level glutamate distribution. However, like Larkin et al. [27] and Zhai et al. [28], our results are consistent with the idea that cell-level heterogeneity is important. In our model, a particular amount of variation in propensity to signal is necessary to achieve synchronized oscillations. In the presence of such variation, the cells with the highest propensity to signal hyperpolarize first. Once enough cells participate, a wave of signaling occurs, relieving glutamate stress and suppressing further signaling. Under this hypothesis, the fraction of participating cells settles near the level predicted by percolation theory not because the system explicitly follows percolation dynamics, but because once enough cells signal to relieve stress, further signaling becomes unnecessary.

The observation that a requisite level of variation in signaling propensity is necessary to produce coordinated waves of signaling in our model raises further questions. What could be the source of variation in signaling propensity, and how could this variation be maintained? *In vitro* biofilms observed to participate in signaling are typically clonal, so variation in signaling behavior is unlikely to be genetic in well-studied cases. Yet signaling behavior is observed to be heritable, in the sense that daughter cells are more likely to participate in signaling waves if their mother cells signal. One speculative possibility is that the regulatory network controlling potassium channel expression [44] results in multi-generational epigenetic inheritance of signaling [9,45]. Heterogeneity in potassium channel gating may also affect signaling propensity [21].

Whatever the source of the variation, on the basis of current observations, if the apparent individual-level cost of signaling is in fact an artifact of ThT staining [43], cells with a proclivity to signal might be expected to increase in frequency within the biofilm, taking up more glutamate than their neighbors, dividing more quickly, and potentially transmitting (non-genetically) their elevated propensity to signal to their offspring. Depending on how propensity to signal is realized and transmitted, such a process could lead to a decline of variation in propensity to signal, or at least to a decline of heritable variation, if continued long enough and if there are no forces generating new heritable variation (analogous to mutation). (Our model contains such a force, as random deviations from a parent cell’s signaling threshold are partially inherited by offspring.) In our model, if too many cells signal, oscillations collapse and much of the biofilm starves. Thus, our model raises a possibility that is almost the reverse of the one raised by Larkin et al. [27] and Zhai et al. [28]—if signaling improves glutamate uptake for the signaling cell and reduces glutamate uptake for its neighbors, we might think of the cells that do not signal, rather than the ones that do, as acting altruistically, giving up their access to glutamate so that signaling does not collapse. There remain other possibilities—there may in fact be a cost of signaling for the individual, the increase in glutamate uptake from signaling may be dependent on the signaling state of a cell’s neighbors, or any of a number of others. In our current implementation, reproduction is not dependent on internal glutamate, so we do not explore such questions, but they are important for future theoretical and experimental work.

Our interest here is in modeling biofilms within flow cells, which are only a few layers of cells high and thus effectively two dimensional, especially at the edge where signaling behaviors have been most closely studied [27,46]. This near-two-dimensional structure is convenient for models like ours and also convenient for observation of signaling behavior by ThT staining. However, in many more realistic ecological situations, biofilms live in three dimensions, growing in all directions and forming themselves around root systems and other complicated structures [34]. Future versions of models like ours could explore the effects of this three-dimensional structure, potentially informing experimental design in settings in which ThT signal tracing is infeasible. Three-dimensional structures will plausibly influence the effects of signaling and the fraction of signaling cells, since cells in the interior of a three-dimensional biofilm have neighbors in all directions.

Another area of future study involves extending our model to predict how other processes are altered by emergent electrochemical signaling. For example, the expression of some genes has been proposed to be regulated by ion-responsive kinases [47]. By coupling cellular potassium flux to gene expression in our model, we could predict patterns of gene expression heterogeneity that would arise due to signaling. In addition, other cell phenotypes are regulated by nutrient conditions, notably matrix production and sporulation [48]. By modeling the response of genetic circuits that control the differentiation into these phenotypes [49], we could predict how the altered distribution of nutrients in signaling biofilms in turn alters the distribution of matrix producers and spores [10,50,51]. Our model may prove valuable to understanding the feedback between cellular phenomena and emergent nutrient conditions within biofilms, a topic of recent interest [52].

Overall, our work shows that combining agent-based and diffusion-based models can account for the emergence of community-level properties from interactions of individual cells. Doing so allows us to study the effect of signaling behavior on the biofilm as a whole, and on individual cells, taking into account heterogeneity among cells. That so many of the collective and cell-level signatures of *B. subtilis* biofilm signaling can be observed in a simple model hints at a relatively simple set of principles governing *in vitro* signaling behavior.

## Materials and methods

### Model development

Our model is a network agent-based model, where cells are simulated as individual “agents,” each with their own set of rules for interacting with each other and their environment. Cells are placed on a network, where each cell is on a node and can interact with its neighbors. In the context of biofilms, neighbors are adjacent cells. During each unit of time (a “tick,” representing 1.2 minutes in this model) every cell performs actions according to their governing equations, and the environment is updated. We model the biofilm as hexagonal, matching observations by Larkin et al. [27] that cells in these biofilms have a modal value of 6 immediate neighbors.

To determine which interactions to include and how cells should behave, we followed the model from Martinez-Corral et al. [29]. Their model is an ODE system describing a one-dimensional cross-section of a *B. subtilis* biofilm. We simplify their equations to be tractable for an agent-based model, leaving us with 4 equations (Eq S1, Eqs S3-S5) that describe potassium uptake and release, glutamate uptake and consumption, membrane potential, and the interactions between potassium, glutamate, and membrane potential.

#### Initialization and growth.

To initialize the model, we “grow” the biofilm, drawing each layer from the previous one. We begin by making a hexagon of 7 cells (6 outer and one center cell). These have signaling thresholds (the level of internal glutamate they can drop to before they will signal) randomly drawn from a uniform between 0 and 3. We then grow the biofilm to a radius of 40 cells while all external variables remain static: we ignore diffusion, metabolism, and signaling during this period. Each tick we randomly select one-fortieth of the cells on the perimeter of the biofilm network, with replacement, to reproduce. Each daughter cell (*j*) is a clone of its parent (*k*), except that its signaling threshold is drawn from a truncated normal with bounds of 0 and 3 in most of the work reported here, and with *σ* of 1 and mean equal to the parent’s signaling threshold. The cell is placed in one of the empty nodes adjacent to the parent, with probability proportional to the number of neighboring cells each empty node has.

Once this initial phase of growth is complete, we begin to simulate potassium and glutamate behavior. Each tick, we update potassium via Eq S4, simulating absorption, signaling, and diffusion. Simultaneously, we update glutamate via Eq S1 and Eq S3, simulating metabolism and absorption and using the algorithm described in S1 Text Sect 3.1 to approximate diffusion. We make the assumption that half the volume of the biofilm is contained within cells and half is extracellular media, so concentration change can be calculated without scaling. We calculate the change in membrane potentials for each cell based on the results from the potassium calculations (Eq S5). We continue growth at a rate of one-fortieth of the perimeter per tick until the network reaches a population of approximately 51,000 cells.

### Model validation

We validated our model by replicating previous experiments by other researchers. As a control, we ran the model 20 times under default conditions (using the parameters given in S2 Tab). Each run recorded a variety of data, with 5 of the runs recording individual signaling and glutamate data for every cell during each tick. These runs were used to gather summary statistics including signaling rate, recurrence rates and growth trajectories.

#### Perturbations.

To test the effect of increased glutamate, basal glutamate was increased to 35 mM from 30 mM for 200 iterations in a biofilm that had already been growing for 3100 iterations. To test potassium shock, we increased basal potassium from 8 mM to 300 mM for 5 ticks in a growing biofilm, beginning at 1100 ticks. We also simulated a biofilm with basal glutamate at 20 mM, limiting its growth to a radius of approximately 90 cells. The results from these perturbations are shown in Fig 4.

#### Oscillation synchronization.

To explore the effect of extracellular potassium to trigger oscillation synchronizations, we first calculated the approximate diffusion of potassium out of the biofilm (see S1 Text Sect 3.5). This calculation produced an estimated change in basal potassium for a neighboring biofilm at a distance of 2 mm. We then applied this oscillation in basal potassium to 20 simulations of stably oscillating biofilms.

This was repeated every 100 μm from 350 to 4000 μm. To assess whether oscillations across each replicate were synchronized, we developed a metric to determine the range of variation. Between ticks 600 and 750 after potassium oscillations began, we recorded the times where signaling was at a minimum for each replicate. Then, for each of the 4 oscillations over that time, we calculated the interquartile range of minimum signaling times across our 20 replicates. The reported “Asynchrony Index” values in Fig 5 and S6 Fig were the average of these ranges, scaled by the mean range when neither potassium nor glutamate were being externally oscillated (the null range).

#### Glutamate diffusion.

Explicitly including glutamate diffusion would have been computationally intractable in two dimensions and over the timescales we wished to simulate. Instead, we used an approximation described in S1 Text Sect 3.1. We validated this approximation by simulating the biofilm with glutamate diffusion and absorption, but no signaling, and comparing the glutamate concentration in each layer of the biofilm with that produced by an explicit model of one-dimensional glutamate diffusion, described in S1 Text Sect 3.6. The resulting concentrations of glutamate with depth (shown in S8 Fig) are broadly similar, with a discrepancy near the boundary between inner and outer cells, where glutamate decreases more rapidly in our heuristic approximation than in the explicit model.

### Experiments

Biofilms experiments were performed in a microfluidic device (CellASIC ONIX2 B04-F plate, Millipore Sigma, Burlington, MA, USA) as described in previous work [21,27]. Cells (*Bacillus subtilis* strain NCIB3610, Bacillus Genetic Stock Center) were streaked on LB agar plates, incubated overnight at 37°C, grown in liquid LB medium, resuspended in liquid msgg medium for additional growth, and loaded into the microfluidic plate. The composition of msgg was 5 mM potassium phosphate (pH 7.0), 100 mM MOPS (pH 7.0), 2 mM MgCl_2_, 700 μM CaCl_2_, 50 μM MnCl_2_, 100 μM FeCl_3_, 1 μM ZnCl_2_, 2 μ M thiamine HCl, 0.5% (v/v) glycerol and 0.125% (w/v) monosodium glutamate. After cell loading into the microfluidic plate, biofilms were grown under flow at 30°C and Thioflavin-T (ThT) was added to the media for imaging cellular membrane potential after 12 hours of growth [21]. Biofilms were imaged in phase contrast and fluorescence with a 4X, 0.13 NA objective on an Olympus IX-83 microscope (Evident Scientific, Waltham, MA, USA).

Time traces of ThT were extracted from time-lapse movies using a machine learning-based segmentation approach implemented in Python, which applies a random forest classifier, provided by the Scikit-learn library, trained on manually segmented biofilm images to perform segmentation using the ThT fluorescence channel. In the ThT traces of Fig 3, we subtracted slow accumulation of ThT *post hoc* to make oscillation traces stationary.

Pairwise signaling consistency calculations given in Table 1 and S1 Tab were calculated by tracing the signaling states of approximately 300 cells across a 2 hour period that included two oscillations.

## Supporting information

S1 Text Supplementary text providing detailed information on methodology and modeling. (PDF)

S1 Fig Time-series data for a signaling cell in the periphery of the biofilm. (A) Internal glutamate is initially consumed faster than glutamate uptake can replenish it. Once internal glutamate drops below a threshold value, signaling occurs, and the cell absorbs more glutamate. (B) When signaling occurs, a cell releases potassium, causing internal potassium levels to drop. After that, potassium slowly returns to its set point. (C) The release of internal potassium during signaling causes membrane potential to spike temporarily, facilitating glutamate uptake. Oscillations take about 45 ticks each. Potassium and glutamate are in mM, membrane potential is in mV.
(TIFF)

S2 Fig Distribution of glutamate across the biofilm over different threshold ranges. An extension of Fig 6H and I. Here we show the mean internal glutamate over time for each cell in the biofilm. Dark purple indicates high glutamate, light indicates low. (A) has no signaling. (B) has bounds of [−0.4, 2.5], producing minimal oscillations. (C) is the regime used in our main results—[0, 3]—which produces stable oscillations similar to those observed *in vitro*. And (D) has bounds of [0.3, 2.9], which triggers signaling collapse.
(TIFF)

S3 Fig An illustration of the boundary between the interior and exterior of a biofilm. Images are of 4 different time points in an oscillation. The yellow hexagon is the boundary between interior and exterior. Cyan indicates hyper-polarized cells.
(TIFF)

S4 Fig Distribution of cellular ThT during a signaling peak. The dotted line indicates the cutoff point for signaling cells, defined as the median value plus an adjustment value of 0.35. Cells with ThT greater than this (dark gray) are defined as signalers. Those with one lower (light gray) are non-signalers.
(TIFF)

S5 Fig Comparisons between internal glutamate, signaling thresholds, time spent signaling, and depth within the biofilm. Percentage of time spent signaling is by cell and across all ticks, not just during signaling peaks. For depth within the biofilm, 0 indicates a cell on the edge of the biofilm. The dark line of cells in (B-D) are cells on the exterior of the biofilm, and the scattered lighter cells are interior cells. The black cells at the bottom of (A) and the light gray at the bottom right of (D) are cells at the center of the biofilm that have near-zero glutamate and therefore struggle to signal.
(TIFF)

S6 Fig The effect of oscillating basal glutamate in the media on signal synchronization. 20 biofilms were simulated. After 3000 ticks, we began oscillating external glutamate with a period equal to the period of signaling oscillations, and magnitude of oscillation as shown on the x-axis. Each of our 20 replicates had the same glutamate trajectory. The Asynchrony Index is a measure of how widely spread the simulations are after approximately 600 ticks—0 indicates perfect synchrony and 1 indicates a degree of asynchrony similar to that obtained with no potassium oscillation. Glutamate had an appreciable effect at around 0.5 mM oscillation magnitude.
(TIFF)

S7 Fig The behavior of signaling oscillations for simulations across various signaling threshold ranges. The threshold bounds for each simulation are [x, y], rounded to the nearest 0.1. For each sub-plot, the x-axis is the time (200 ticks) and the y-axis the fraction of signaling cells. Plots in the upper right with no signaling are cases in which the biofilm collapsed and extracellular potassium became extremely high. These data were used to create the phase diagram in Fig 6.
(PDF)

S8 Fig A comparison between the heuristic simulation of glutamate used in our model and a physically accurate simulation. We removed cell signaling but retained glutamate uptake and degradation; the y-axis indicates mean internal glutamate in cells (results from a single simulation, which is effectively deterministic with these modifications). The x-axis is the distance from the center of the biofilm (in units of cells), with 0 being the center and a border at approximately 130 cells. The heuristic model shows a more abrupt drop-off in internal glutamate.
(TIFF)

S1 Video *In vitro B. subtilis* biofilm signaling oscillations. This is a time-lapse of microscope images of a *B. subtilis* biofilm exhibiting the oscillatory behavior *in vitro*. The biofilm has been stained with the fluorescent membrane potential reporter ThT; cyan indicates hyperpolarized cells. The scale bar is 100 μm.
(GIF)

S2 Video Simulated B. subtilis signaling oscillations. This is a time-lapse of our simulated model. Here too, cyan indicates hyperpolarization. This video loops after five oscillations.
(MP4)

S1 Table An extended version of Table 1. Values for inner and total cells have been added in addition to outer cells. We also include consistency and inheritance estimates for the entire duration of signaling, not just pairwise, and with signaling measured across an entire oscillation, not only during the peak of signaling. Errors for all simulated results are standard deviations. For the signaling fraction and pairwise recurrences, standard deviations are estimated using 20 runs. For the rest, they are estimated using five runs. Error for the observed signaling fraction is a standard error from Zhai et al. [28]. Errors for observed signaling consistency are standard errors estimated as for a binomially distributed observation (number of cells = 316). (Standard error values were not reported in Zhai et al. [28]. We estimated them based on the given recurrence rates (0.6 and 0.78), fraction of signalers (43%), and number of observations (49 pairs of cells). This suggested 22 initial signalers, of whom 13 have signaling offspring, and 27 non-signalers, of whom 21 have non-signaling offspring. This produced the standard errors given above assuming binomially distributed counts.)
(PDF)

S2 TableDefault values and units for all parameters used in the model.Content, formatting, and descriptions closely follow those in Martinez-Corral et al. [29]. The parameter that was labeled *D*_*p*_ in their paper has been changed to $\alpha_{k}$. Parameters divided by 50 were originally in units of hours but have been converted to be in units of ticks (t).(PDF)
