# Supplementary material for: An agent-based model of metabolic signaling oscillations in Bacillus subtilis biofilms
Source: PLoS Comput Biol. 2025 Dec 4;21(12):e1013746. doi: 10.1371/journal.pcbi.1013746 (PMC12694845; doi:10.1371/journal.pcbi.1013746)
Supplement: S1 Text — (PDF) [file pcbi.1013746.s001.pdf]

Supplementary text for ”An Agent-Based Model of Metabolic Signaling Oscillations in *Bacillus subtilis* Biofilms”

Obadiah J. Mulder[1], Maya Peters Kostman[2], Abdulrahmen Almodaimegh[2], Michael D. Edge[1], and Joseph Larkin[2]

[1]Department of Quantitative and Computational Biology, University of Southern California, Los Angeles, CA, USA

[2]Departments of Biology and Physics, Boston University, Boston, MA, USA

October 18, 2025

1 Definitions

1.1 Signaling

The phenomenon we refer to as “signaling” occurs via membrane polarization. Larkin et al. [4] observed that cell polarization during a “wave” of signaling is bimodal, with cells that have recently released potassium having high polarization and other cells having lower polarization. In our model, scaled ThT (a stain representing membrane potential) is bimodal, with a distribution similar to that observed by Larkin and colleagues. We define signaling cells as those belonging to the higher peak, with internal ThT greater than the median value for scaled ThT plus a small factor (0.035). The entire distribution shifts depending on the phase of the signaling oscillation; this definition accounts for that. An illustration of the distribution of ThT and the cutoff for signaling is given in Fig S4.

1.2 Interior and exterior cells

Both our simulations and *in vitro* observations show that the interior and exterior of the biofilm behave differently from each other (Fig 3). We track behavior for these two groups of cells separately in addition to tracking biofilm-wide indicators. The boundary between inner and outer cells is not determined visually, nor by signaling behavior. Instead, we observed that cell behavior changes qualitatively at approximately the depth to which glutamate diffuses if there is no signaling. To determine the depth from the exterior of the biofilm to the boundary, we thus set cell membrane potentials to be the default of -86 mV, internal potassium to its default of 300 mM, and external potassium to the basal level of 8 mM. We then applied our algorithm for determining glutamate diffusion and set all cells that received any glutamate as exterior. We defined interior cells as those at least 8 cells in from the boundary at which cells receive no glutamate. The intervening 8-cell layer was classed as neither interior or exterior. In practice, the boundary of the exterior produced by this method is somewhat closer to the exterior of the biofilm than the qualitative boundary between inner and outer cells. This approach is appropriate to ensure consistency for our summary statistics of outer cells with those from prior studies that focused solely on the biofilm's exterior.

2 Changing the heritability of the signaling threshold

Changing the standard deviation of the truncated normal from which signaling thresholds were drawn changed the heritability of signaling behavior. The lower the standard deviation $\sigma_{\mathcal{T}}$, the more heritable the signaling threshold. If $\sigma_{\mathcal{T}}$ was reduced too much, then the distribution of thresholds across the biofilm became uneven and strongly dependent on the initial draws of signaling thresholds at the foundation of the simulated biofilm. In this regime, signaling usually occurred either at a very low rate or the biofilm collapsed. If $\sigma_{\mathcal{T}}$ was raised to 2, then we still observed oscillations of ap-

proximately the same magnitude as if $\sigma_{\mathcal{T}}$ equals 1, but the signaling recurrence rates decreased appreciably, as might be expected. The daughters of signaling cells signaled 49 $\pm$ 0.2% of the time, as opposed to 63 $\pm$ 0.3% when $\sigma_{\mathcal{T}} = 1$ and 60% in the *in vitro* observations of Zhai et al. [15]. For non-signaling cells, daughter cells' frequency of non-signaling behavior dropped from 73 $\pm$ 0.2% to 63 $\pm$ 0.1%, as opposed to 78% *in vitro*.

3 Model description

Our model was built on the equations in the following subsections, adapted from Martinez-Corral et al. [8]. Martinez-Corral and colleagues modeled glutamate and potassium diffusion, absorption, release, and metabolism. They also modeled glutamate membrane transporter density, ammonium (which affects the rate of decay of intracellular glutamate) and GDH enzyme (which affects the production of ammonium). The price of using an agent-based model is that simulations are slower and more computationally expensive, so we applied some simplifications. We modeled glutamate, potassium, and membrane potential, but we did not model transporters, ammonium, or GDH. Instead we set those parameters to the default values from Martinez-Corral et al. [8]. The parameter values for these equations can be found in Table S2. Further, for the factors we did explicitly model, we closely followed the approach in Martinez-Corral et al. [8].

3.1 Glutamate

Internal glutamate (G_i) is the amount of glutamate within each cell. Each tick, we calculated glutamate absorption (affected by the uptake constant α_g, the membrane potential V, and the amount of external glutamate G_e). Internal glutamate is consumed at a constant rate δ_g. α_g and δ_g were fit to produce expected behavior (see S1 Text section 3.4.1 for details on this process). The rate of glutamate uptake increases asymptot-

ically with extracellular glutamate; k_g is the concentration of extracellular glutamate at half-maximal uptake rate. This value was fit by Martinez-Corral et al. [8]. We assumed that cells do not release glutamate, and so the rate of change of internal glutamate is the rate of uptake minus consumption,

$$\Delta G_i = \alpha_g \mathscr{F}(V) \frac{G_e}{k_g + G_e} - \delta_g G_i \tag{S1}$$

where

$$\mathscr{F}(V) = \frac{1}{1 + e^{V - V_0}}. \tag{S2}$$

Here $\mathscr{F}(V)$ represents the logistic effect of membrane potential on glutamate uptake. If a cell is polarized, then there is minimal hindrance to uptake, but once it becomes depolarized, glutamate uptake rapidly goes to zero. This equation was also taken from Martinez-Corral et al. [8].

External glutamate for each cell (the amount of glutamate outside of the cell, used to determine glutamate uptake) is affected by glutamate uptake, which moves external glutamate to the cell's interior, and by diffusion. To make our model computationally tractable, we modeled glutamate diffusion via a simplified heuristic approximation. See S1 Text section 3.6 for more details on the performance of the heuristic approach and our reasons for employing it. We assume that glutamate diffuses exclusively from outside the biofilm through to the middle, with no diffusion perpendicular to this direction (i.e. no tangential diffusion around the 2D biofilm). To simulate diffusion, we thus calculated glutamate diffusion from each layer of the biofilm to the next, beginning at the exterior and working inwards. For perimeter cells, we set the level of available glutamate G_e to the basal concentration of glutamate in the media. We then calculated glutamate absorption for perimeter cells and subtracted this from the available glutamate. The remaining glutamate was then used as the available glutamate when calculating absorption for the next layer. This process was repeated layer by layer moving inward.

Note that this was performed at the resolution of individual cells, for each cell averaging the available glutamate remaining from the two adjacent cells in the previous layer. So for a cell j with two neighbors in the previous layer k and l, the external glutamate available to j to absorb would be

$$G_{ej} = \text{mean}\left(G_{ek} - \alpha_g \mathscr{F}(V)\frac{G_{ek}}{k_g + G_{ek}},\ G_{el} - \alpha_g \mathscr{F}(V)\frac{G_{el}}{k_g + G_{el}} \right), \tag{S3}$$

and the glutamate available to cells interior to j would be $G_{ej} - \Delta G_{ij}$. In this way, glutamate approximately diffuses to the biofilm center each tick, but is allocated among cells radially, albeit maintaining some centripetal spatial effects by the transmission to two immediately adjacent cells in the next layer. Thus, the glutamate available to a cell is effected by the glutamate absorption of all cells exterior to it in a 60-degree cone extending to the edge of the biofilm.

3.2 Potassium

In our model, internal potassium concentration is governed by potassium uptake and by potassium release that occurs during signaling. Potassium uptake is proportional (via a fit constant α_k) to the product of internal glutamate, external potassium, and the difference between a homeostatic set point for potassium (K_{i0}) and the cell's current internal potassium (K_i). (Note that we labeled this process "uptake," but it can reverse in principle if $K_i > K_{i0}$.) This assumes cells try to maintain a constant level of potassium, with glutamate included to represent the energy requirements for potassium absorption.

When a cell signals (encoded below by the indicator variable $\mathcal{I}_{\{\mathcal{T}\}}$; when a cell is signaling, $\mathcal{I}_{\{\mathcal{T}\}} = 1$, otherwise $\mathcal{I}_{\{\mathcal{T}\}} = 0$), it releases a large amount of potassium, with the amount depending on the levels of interior and exterior potassium. (Generally there is much more potassium inside the cell than outside, and cells with more internal

potassium, relative to external potassium, release more potassium when signaling.) The amount of potassium released also depends on the membrane polarization of the cell, V; less polarized cells will release more potassium. Combining uptake with outflow during signaling, the change in internal potassium is given by

$$\Delta K_i = \alpha_k G_i K_e (K_{i0} - K_i) - F g_K \left(V - V_{K0} \ln \frac{K_e}{K_i} \right) \mathcal{I}_{\{\mathcal{T}\}}, \tag{S4}$$

where F is the membrane capacitance, g_K is the potassium channel conductance, and V_{K0} is the Nernst potential prefactor.

Like glutamate, external potassium is determined by the uptake and release of potassium from a cell, plus diffusion. Unlike glutamate, potassium is a small ion and diffuses rapidly with respect to the coarse discretization of time we employed [13, 10]. Further, potassium has a much more even distribution across the biofilm than glutamate does. Thus, to simulate diffusion, we computed per-cell external potassium levels by accounting for uptake and signaling via Eq S4, and then averaged external potassium across all cells in the biofilm every tick.

3.3 Membrane potential

When a cell is not signaling, the membrane potential approaches a set point $V_{L0} = -93.5$ mV, but can become more positive (less polarized) if external potassium is close to or greater than basal potassium in the media (K_m). During a signaling event, membrane potential becomes much more negative (polarized) in proportion with the amount of potassium released. Following Martinez-Corral et al. [8], we modeled changes in membrane potential as

$$\Delta V = -g_L \left(V - \left(V_{L0} + d_L \frac{K_e - K_m}{1 - e^{(K_m - K_e)/\sigma}} \right) \right) - g_K \left(V - V_{K0} \ln \frac{K_e}{K_i} \right) \mathcal{I}_{\{\mathcal{T}\}}. \tag{S5}$$

This expression is a discrete version of equation S18 of Martinez-Corral et al. [8]—modeled assuming Hodgkin–Huxley-like conductance mechanics—with the signaling indicator variable $I_{\{T\}}$ replacing a variable indicating propensity to signal. g_L is the leak conductance and d_L is the leak slope coefficient.

3.3.1 ThT staining

In order to replicate *in vitro* studies that used ThT staining to detect cells with extreme membrane potentials, we simulated ThT absorption.

$$\Delta T = \alpha_T \left(1 + e^{g_T V - V_{0T}}\right)^{-1} - \gamma_T T \tag{S6}$$

This equation is a discretized version of equation S20 of Martinez-Corral et al. [8]. α_T is the maximal rate of ThT uptake, g_T is the inverse sensitivity of ThT to membrane potential, and γ_T is a decay constant. The ThT fluorescence threshold V_{0T} represents the membrane potential at which a cell absorbs ThT at half the maximal rate.

3.4 Parameters

In a previous version of this manuscript [9], we used a Nernst potential prefactor (V_{K0}) of 100 mV, inherited from previous modeling papers in *B. subtilis* biofilms [7, 8, 10]. An anonymous reviewer pointed out that this is not a free variable and the correct value is 25.8 mV at 300 Kelvin. From this, V_{L0} can be calculated to be -93.5 mV given the default values for internal and external potassium used in our model (see [14] for a breakdown of this calculation). We used these corrected parameters in our model here. We set *B. subtilis* resting membrane potential to -86 mV. The exact value is unknown, but for the purposes of our model it must be closer to zero than V_{L0}, and -86 falls within previous estimates of -65 to -110 mV [3, 11, 14].

Most other parameters were taken from Martinez-Corral et al. [8]. We chose val-

ues of δ_g, α_k, F, g_k, and d_L that were near previously used values for these parameters—all free variables and, to our knowledge, not empirically estimated—and that produced oscillations similar to those observed *in vitro* using the corrected Nernst potential prefactor of 25.8 mV (see Table S2). This process of setting the parameters to levels that produced oscillatory behavior was also followed in other papers. In our case, this process was able to produce oscillatory behavior similar to what we originally obtained with the Nernst potential prefactor of 100 mV, and similar to that of other models that used this value. To the extent the results of the models hinge on cell physiology, past models using the value of 100 mV should be reevaluated for more realistic parameter values. For basal nutrient concentrations, the values were set to be equal to those used by Martinez-Corral et al. [8].

3.4.1 Parameter fitting

Fit parameters were adjusted to produce behavior similar to *in vitro* oscillations. In particular, we selected parameters that produced oscillations with a minimum fraction of signalers near zero and a maximum fraction near 43%. A subset of parameters also produced a large core of starved cells with near-zero glutamate at the center of the biofilm and we selected parameters to minimize this effect. Whether these effects were produced was assessed visually, by plotting the distribution of glutamate in the biofilm, and the fraction of signaling cells over time.

3.4.2 Scaling

Our model is in units of ticks, while Martinez-Corral et al. [8] used hours (as have other ODE-based approaches). We aimed to set ticks to last 1.2 minutes and based the growth rate and parameter values off of this. Thus all parameters in units of hours were scaled by a factor of 50. However, in our model, tick size does not change the rate at which glutamate diffuses because we assumed that it diffuses approximately to the center of the biofilm each tick. This assumption was used to make the model

computationally tractable. But it actually takes around 4.1 minutes for glutamate to diffuse to the center of the biofilm ([13] and our own calculations), inducing a discrepancy with our other assumptions. To account for this, we adjusted the parameters dictating potassium uptake α_k and membrane capacitance F (by factors of 2.35 and 4.35 respectively). The fact that glutamate diffusion is slower than we assumed may account for the faster-than-expected oscillations displayed by our model, which have a period of approximately an hour, as opposed to approximately two hours on average *in vitro*. Increasing the tick size to match the 4.1 minutes it takes glutamate to diffuse seemed to be too coarse for the model to tolerate, and changes in many of the variables between ticks become large, leading to unstable behavior. While developing the present model, we pursued another, more computationally intensive approach that involved simulating glutamate and potassium diffusion explicitly, giving some behaviors that matched the current results and oscillation periods closer to *in vitro* observations (results not shown). We opted for the current model for its simplicity and lower computational burden.

3.5 Potassium diffusion and oscillation synchronization

Part of our model validation was to show that the oscillations in potassium outside of a biofilm caused by signaling within a biofilm could trigger synchronization of oscillations in a neighboring signaling biofilm. We used a diffusion constant of $D = 7 \times 10^6$ μm/hr for the rate of potassium diffusion in media. We assumed that the diffusion constant within the biofilm was one quarter of this, following estimates from Stewart [13].

We estimated diffusion discretely, with a time step of 10^{-8} hours. Each tick, we set the potassium (outside of cells) for the region of our biofilm (150 cell radius) to be equal to that from our simulations. We then calculated diffusion for the tick in a circle around our biofilm, assuming concentric layers with a radius of 3 μm (the approximate length of one cell). We recorded the mean external potassium at a distance of 2000

to 2900 μm from the border of our biofilm (approximately the region another biofilm would cover in the experiments from Liu et al. [5]).

To test the effect of this variation in external potassium on our model, we ran 20 simulations using the oscillations in external potassium predicted by our diffusion calculation. The potassium trajectory we calculated includes some build-up of potassium in the media, so the base level of potassium (the lowest values during oscillation) are still above the level in our model. Previous work has suggested that biofilms can adapt to changes in basal potassium, and that the main effect of potassium comes from changes, not constant increases [2, 1]. We thus rescaled the potassium oscillations we calculated so that the minimum value is the basal level of potassium in our model (8 mM). We then used this trajectory as the values of basal potassium (K_m) in simulations. Each tick, we set the basal potassium in the media (K_m) to be the value from our potassium trajectory (scaled so that it began at near magnitude and reached full magnitude after 400 ticks, to replicate a nearby biofilm growing. We then simulated with full-magnitude oscillations for a further 400 ticks. The same potassium trajectory for each simulation.

To determine whether this produced signaling oscillation synchronization, we created an asychrony index. We calculated the range of time-points at which each of the ten simulations reached its minimum signaling level each oscillation, then scaled this range by its mean value in simulations with no synchronization. This value decreases as our simulations synchronize. We repeated this process, calculating the effect of a neighboring biofilm every 0.5 mm from 2 to 4 mm to determine the approximate distance at which signaling no longer produces synchronization. We observed synchronization to largely disappear by 4 mm (Fig 5B).

3.6 Glutamate heuristic accuracy

In our model, we use a simplified method to simulate glutamate diffusion. This was required because our tick size is relatively large compared with the rate at which glutamate diffuses in a biofilm and because our simulated biofilm is highly heterogeneous in the ability of cells to absorb glutamate. Simulating glutamate diffusion in a physically accurate way over two dimensions would be computationally intractable over the timescales we need to model. Although a one-dimension diffusion would be more tractable, it becomes difficult to include the heterogeneity among cells in signaling behavior (and thus glutamate absorption behavior) that is a key part of why we elected to use an agent-based model. However, if we run simulations in our model without signaling, we can compare the effect of our heuristic diffusion to a physically accurate diffusion model and determine whether their behavior is similar.

The diffusion rate of glutamine is 7.6×10^{-6} cm^2/s at 25°C in water (2.7×10^6 μm^2/hr) and the rate in a biofilm is approximately one quarter of this [13]. Satomura et al. [12] found the uptake of glutamine by *B. subtilis* in media with 500 μM glutamine to be between 8 and 12 nmol/min per OD_{600} unit (2×10^{-3} pmols/hr/cell). Zhang et al. [16] also estimated glutamate absorption in *B. subtilis*, putting it between 2.2×10^{-4} and 1.1×10^{-2} pmol/hr/cell. We use a value of 1.94×10^{-3} pmols/hr/cell, in line with this literature.

We modeled diffusion of glutamate across a triangular section of the biofilm. Each layer of our triangle was 3 μm deep (though results are similar with thinner bands), the outermost layer was 131 cells (to match the mean depth of our signaling model), and the number of cells in each layer going inward decreased by one, until the center had a single cell. We allowed glutamate to diffuse from the media at the edge of the biofilm to the center, with a time step size of 10^{-8} hours.

Each time step, we calculated glutamate flux between every pair of layers, as well as glutamate absorption into cells and glutamate degradation by cells. Flux J was calculated as $J = \frac{D_e(c_1 - c_2)}{\delta}$ mM/μm/hr, where D_e is the diffusion coefficient of glutamate in a biofilm in μm^2/hr, c_1 and c_2 were the concentrations of glutamate in each layer in mM, and δ was the thickness of a layer in μm.

We scaled glutamate uptake by $\frac{G_e}{G_e+0.75}$ following Martinez-Corral et al. [8] (G_e being external glutamate in units of mM), set so that maximal uptake was approximately 1.94×10^{-3} pmols/hr/cell. Internal glutamate was consumed following $G_i \times 270 \times$ [time step]. For our time scaling of 10^8 ticks per hour, this came out to $\frac{2.7}{10^6}$ fraction of the available internal glutamate being consumed each tick.

Figure S8 shows how this explicit simulation of glutamate diffusion matches the heuristic simulation used in our model (when signaling is removed). Our heuristic model features a slightly more abrupt drop-off in glutamate, perhaps resulting from a larger time step (i.e. a larger tick size). However, both produce qualitatively similar behavior that is consistent with observations from Liu and colleagues [6] that the interiors of *B. subtilis* biofilms in the conditions we simulate are starved of glutamate.

4 References

[1] Noah Ford et al. "A two-dimensional model of potassium signaling and oscillatory growth in a biofilm". In: *Bulletin of Mathematical Biology* 83 (2021), pp. 1–28.

[2] Jan Gundlach et al. "Control of potassium homeostasis is an essential function of the second messenger cyclic di-AMP in Bacillus subtilis". In: *Science signaling* 10.475 (2017), eaal3011.

[3] Shigeru Hosoi et al. "Control of membrane potential by external H+ concentration in Bacillus subtilis as determined by an ion-selective electrode". In: *Biochimica et Biophysica Acta (BBA)-Biomembranes* 600.3 (1980), pp. 844–852.

[4] Joseph W Larkin et al. "Signal percolation within a bacterial community". In: *Cell systems* 7.2 (2018), pp. 137–145.

[5] Jintao Liu et al. "Coupling between distant biofilms and emergence of nutrient time-sharing". In: *Science* 356.6338 (2017), pp. 638–642.

[6] Jintao Liu et al. "Metabolic co-dependence gives rise to collective oscillations within biofilms". In: *Nature* 523.7562 (2015), pp. 550–554.

[7] Rosa Martinez-Corral et al. "Bistable emergence of oscillations in growing Bacillus subtilis biofilms". In: *Proceedings of the National Academy of Sciences* 115.36 (2018), E8333–E8340.

[8] Rosa Martinez-Corral et al. "Metabolic basis of brain-like electrical signalling in bacterial communities". In: *Philosophical Transactions of the Royal Society B* 374.1774 (2019), p. 20180382.

[9] Obadiah J. Mulder et al. "An Agent-Based Model of Metabolic Signaling Oscillations in Bacillus subtilis Biofilms". In: *bioRxiv* (2024). DOI: `10.1101/2024.12.20.629727`. eprint: `https://www.biorxiv.org/content/early/2024/12/21/2024.`

12.20.629727.full.pdf. URL: https://www.biorxiv.org/content/early/2024/12/21/2024.12.20.629727.

[10] Arthur Prindle et al. "Ion channels enable electrical communication in bacterial communities". In: *Nature* 527.7576 (2015), pp. 59–63.

[11] Debjit Roy et al. "Toward measurements of absolute membrane potential in Bacillus subtilis using fluorescence lifetime". In: *Biophysical Reports* 5.1 (2025).

[12] Takenori Satomura et al. "Enhancement of glutamine utilization in Bacillus subtilis through the GlnK-GlnL two-component regulatory system". In: *Journal of bacteriology* 187.14 (2005), pp. 4813–4821.

[13] Philip S Stewart. "Diffusion in biofilms". In: *Journal of bacteriology* 185.5 (2003), pp. 1485–1491.

[14] J Derk Te Winkel et al. "Analysis of antimicrobial-triggered membrane depolarization using voltage sensitive dyes". In: *Frontiers in cell and developmental biology* 4 (2016), p. 29.

[15] Xiaoling Zhai et al. "Statistics of correlated percolation in a bacterial community". In: *PLoS Computational Biology* 15.12 (2019), e1007508.

[16] Wenbo Zhang et al. "Nutrient depletion in Bacillus subtilis biofilms triggers matrix production". In: *New Journal of Physics* 16.1 (2014), p. 015028.
