# Supplementary figures and images for: An agent-based model of metabolic signaling oscillations in Bacillus subtilis biofilms
Source: PLoS Comput Biol. 2025 Dec 4;21(12):e1013746. doi: 10.1371/journal.pcbi.1013746 (PMC12694845; doi:10.1371/journal.pcbi.1013746)

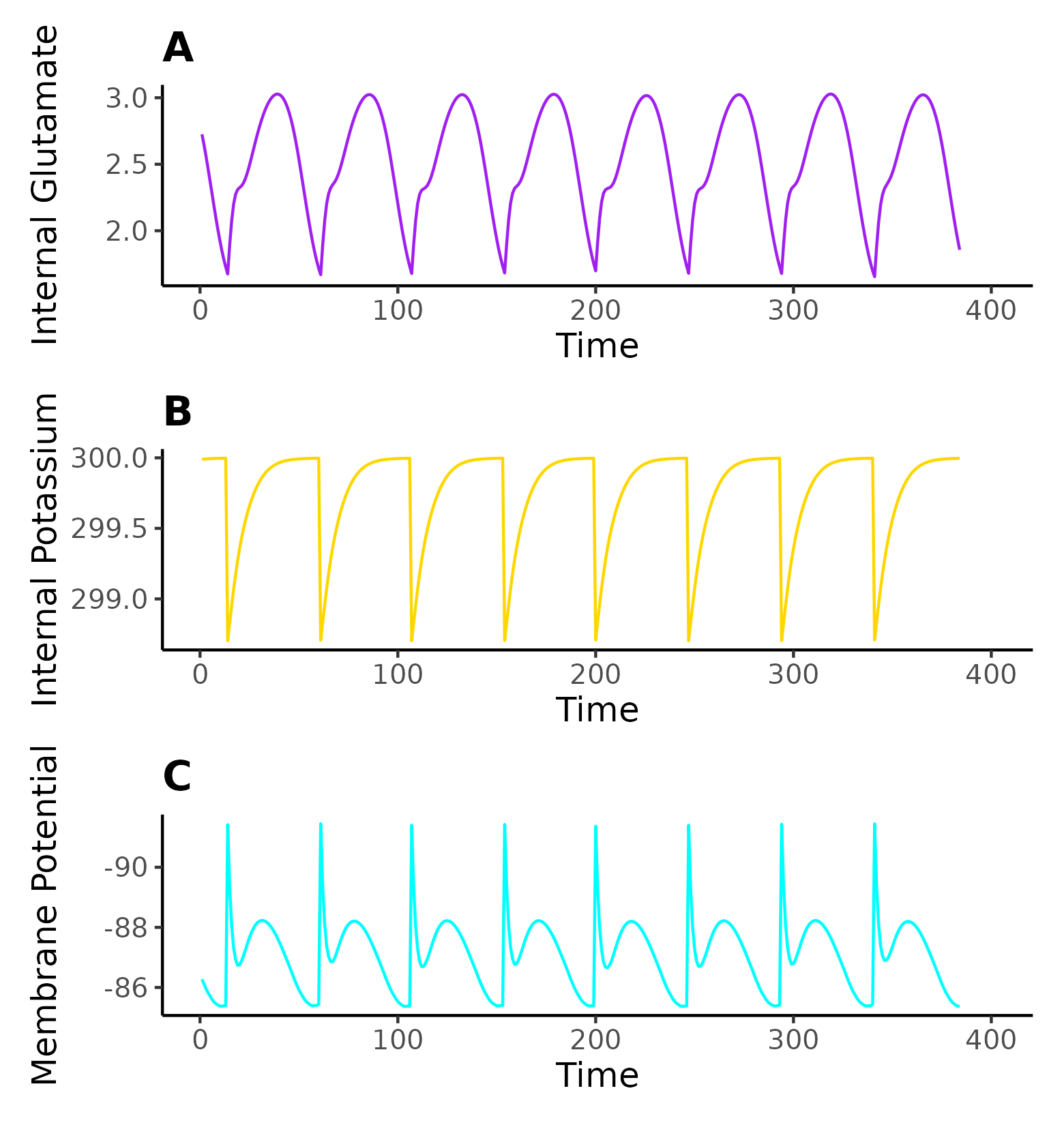

Supplement: S1 Fig — (A) Internal glutamate is initially consumed faster than glutamate uptake can replenish it. Once internal glutamate drops below a threshold value, signaling occurs, and the cell absorbs more glutamate. (B) When signaling occurs, a cell releases potassium, causing internal potassium levels to drop. After that, potassium slowly returns to its set point. (C) The release of internal potassium during signaling causes membrane potential to spike temporarily, facilitating glutamate uptake. Oscillations take about 45 ticks each. Potassium and glutamate are in mM, membrane potential is in mV. (TIFF) [file pcbi.1013746.s002.tif]

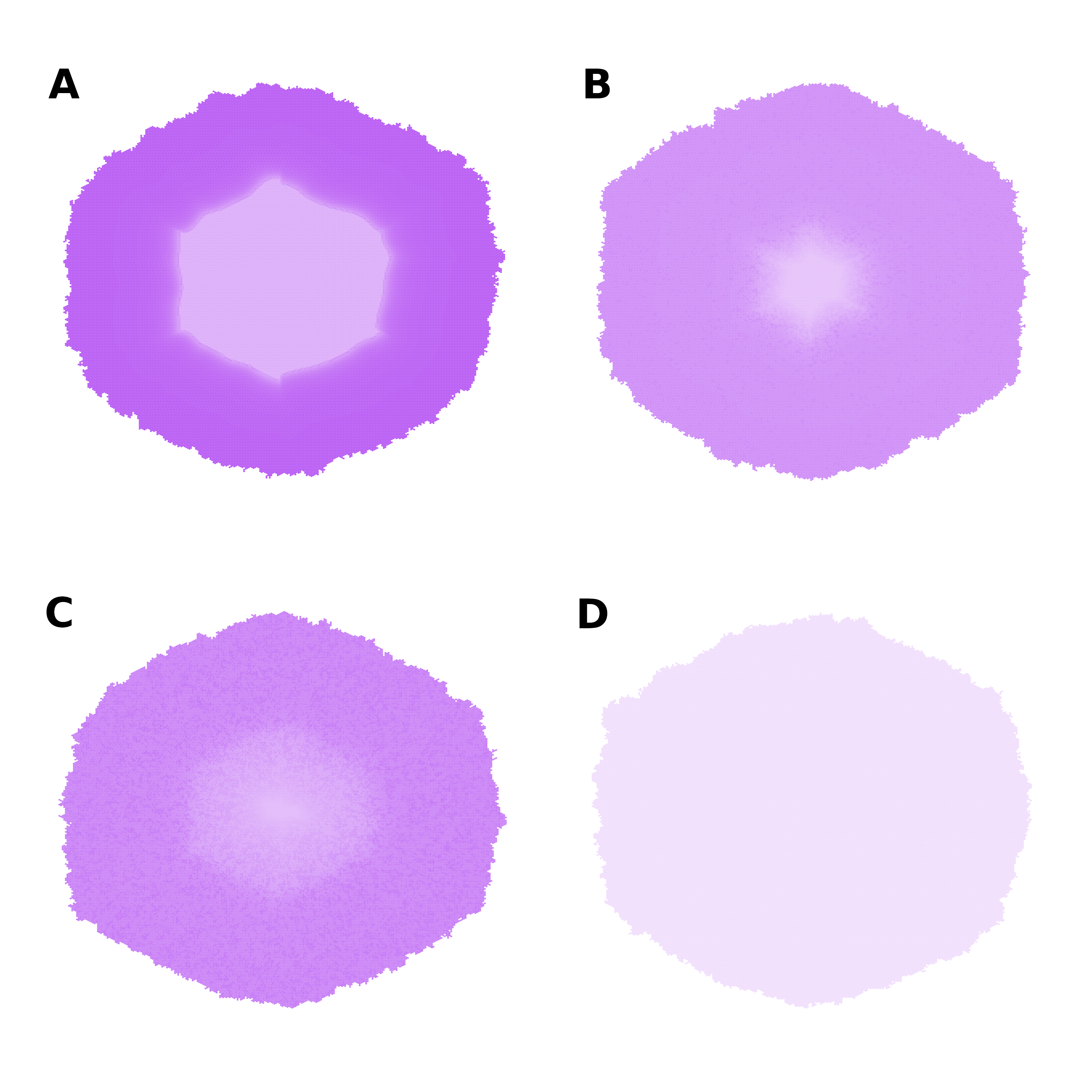

Supplement: S2 Fig — An extension of Fig 6H and I. Here we show the mean internal glutamate over time for each cell in the biofilm. Dark purple indicates high glutamate, light indicates low. (A) has no signaling. (B) has bounds of [−0.4, 2.5], producing minimal oscillations. (C) is the regime used in our main results—[0, 3]—which produces stable oscillations similar to those observed in vitro. And (D) has bounds of [0.3, 2.9], which triggers signaling collapse. (TIFF) [file pcbi.1013746.s003.tif]

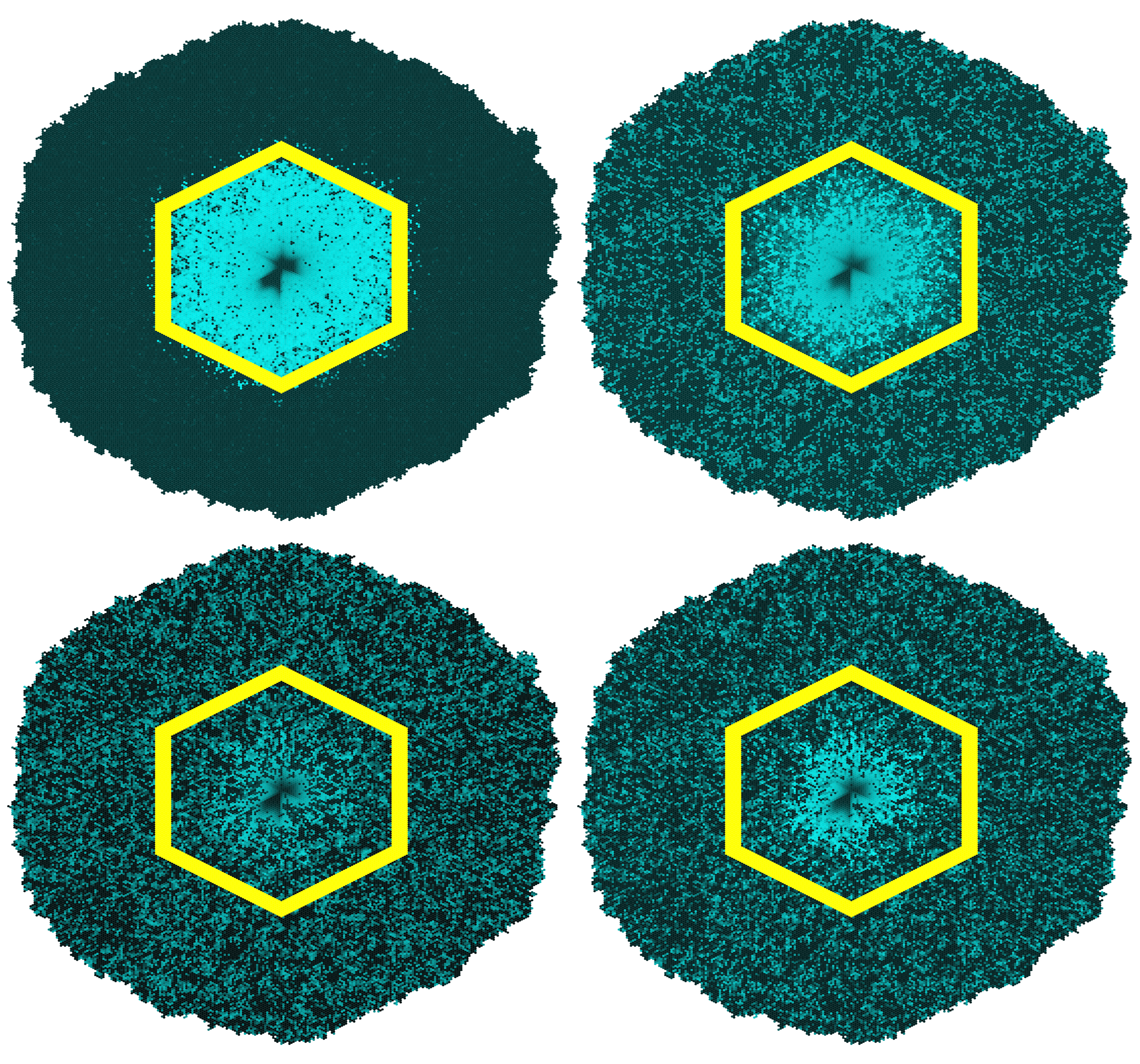

Supplement: S3 Fig — Images are of 4 different time points in an oscillation. The yellow hexagon is the boundary between interior and exterior. Cyan indicates hyper-polarized cells. (TIFF) [file pcbi.1013746.s004.tif]

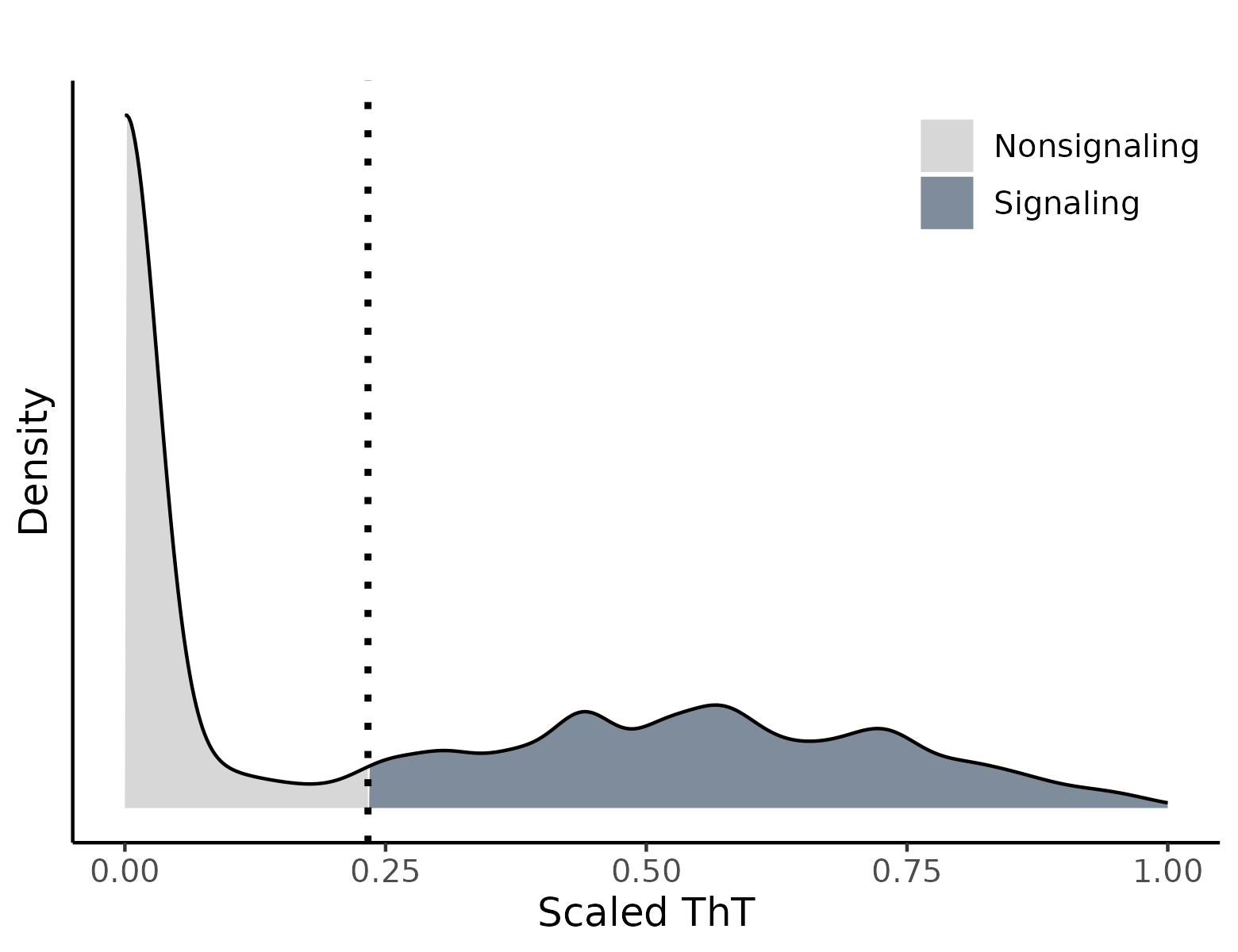

Supplement: S4 Fig — The dotted line indicates the cutoff point for signaling cells, defined as the median value plus an adjustment value of 0.35. Cells with ThT greater than this (dark gray) are defined as signalers. Those with one lower (light gray) are non-signalers. (TIFF) [file pcbi.1013746.s005.tif]

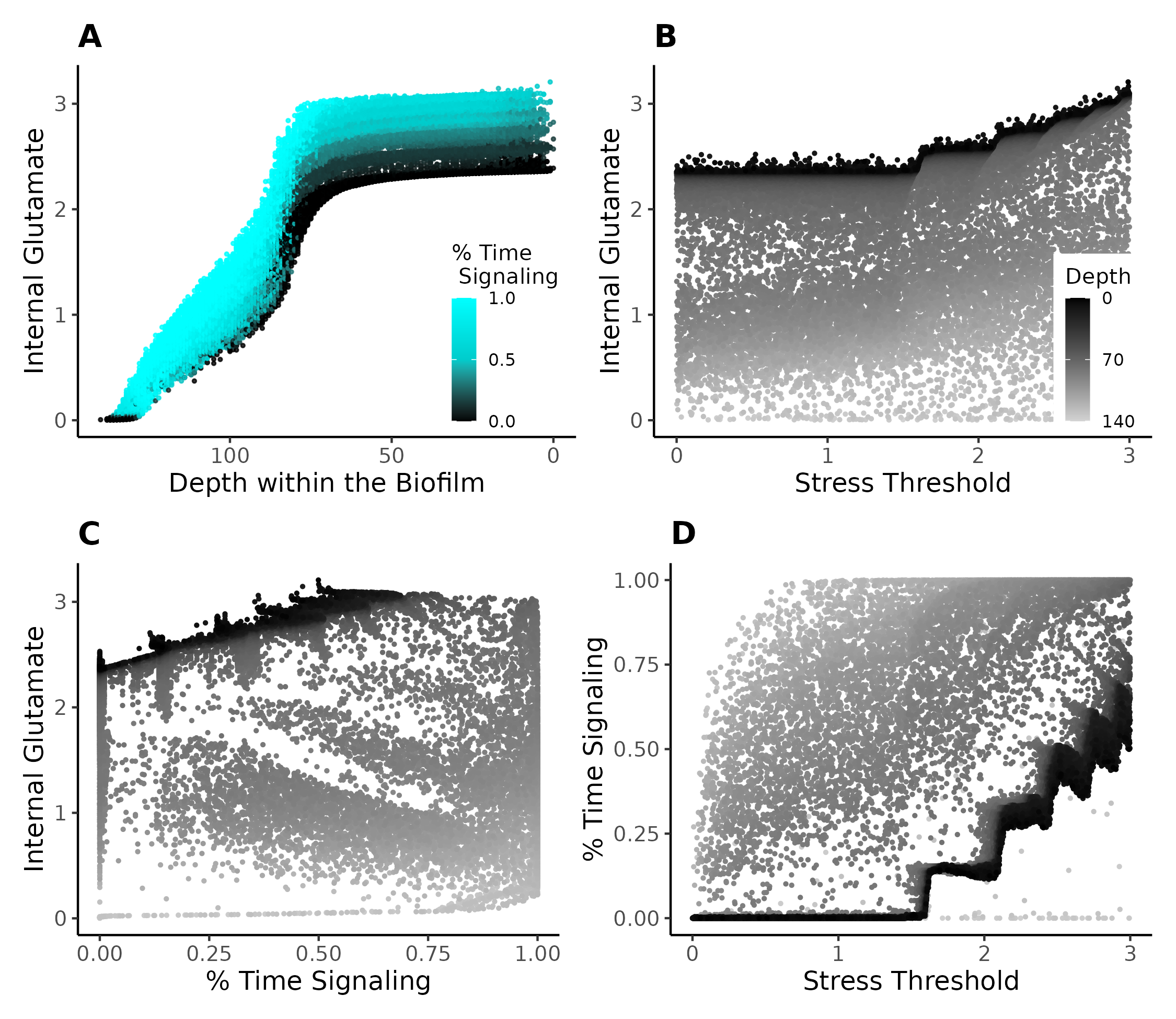

Supplement: S5 Fig — Percentage of time spent signaling is by cell and across all ticks, not just during signaling peaks. For depth within the biofilm, 0 indicates a cell on the edge of the biofilm. The dark line of cells in (B-D) are cells on the exterior of the biofilm, and the scattered lighter cells are interior cells. The black cells at the bottom of (A) and the light gray at the bottom right of (D) are cells at the center of the biofilm that have near-zero glutamate and therefore struggle to signal. (TIFF) [file pcbi.1013746.s006.tif]

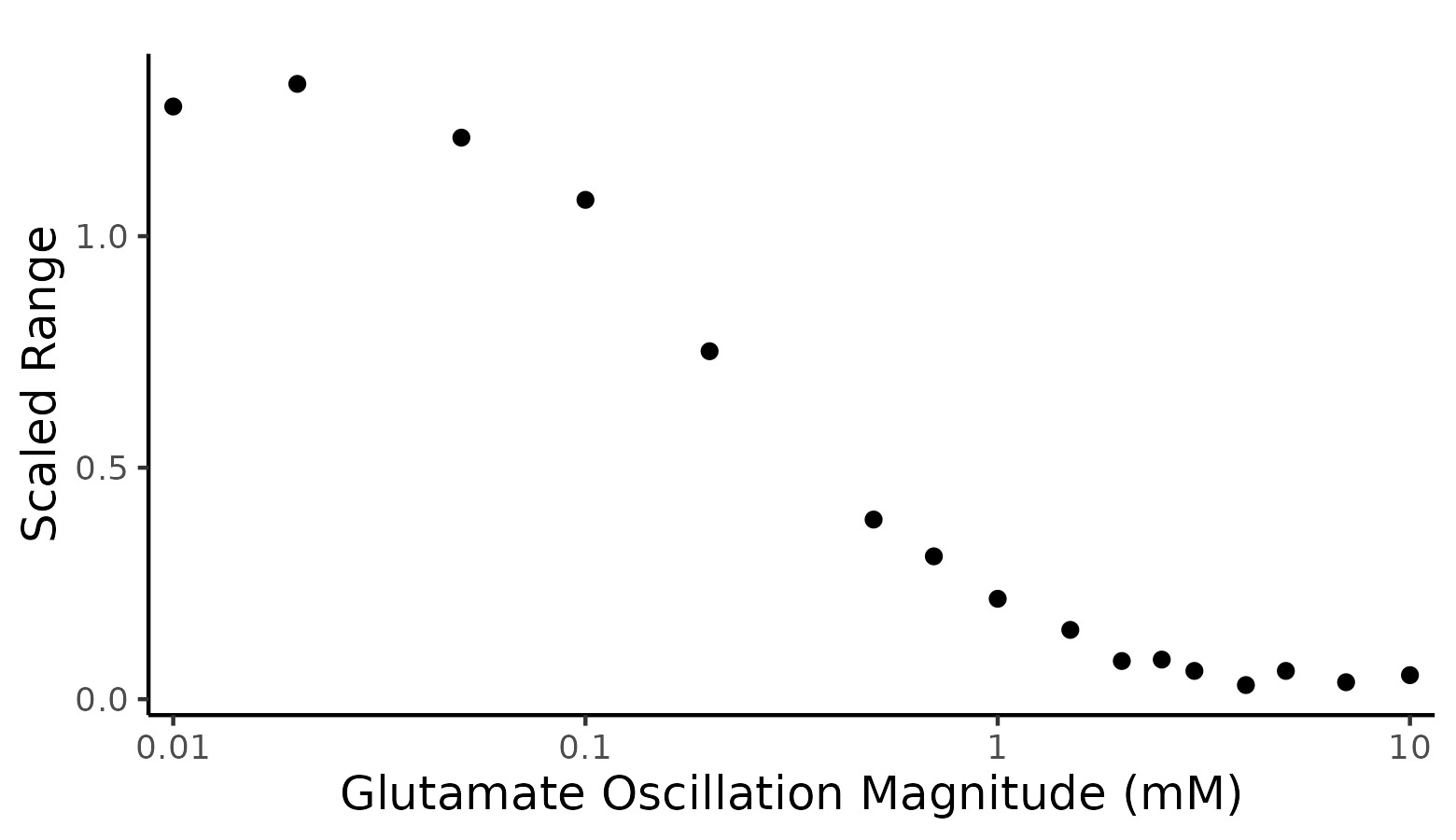

Supplement: S6 Fig — 20 biofilms were simulated. After 3000 ticks, we began oscillating external glutamate with a period equal to the period of signaling oscillations, and magnitude of oscillation as shown on the x-axis. Each of our 20 replicates had the same glutamate trajectory. The Asynchrony Index is a measure of how widely spread the simulations are after approximately 600 ticks—0 indicates perfect synchrony and 1 indicates a degree of asynchrony similar to that obtained with no potassium oscillation. Glutamate had an appreciable effect at around 0.5 mM oscillation magnitude. (TIFF) [file pcbi.1013746.s007.tif]

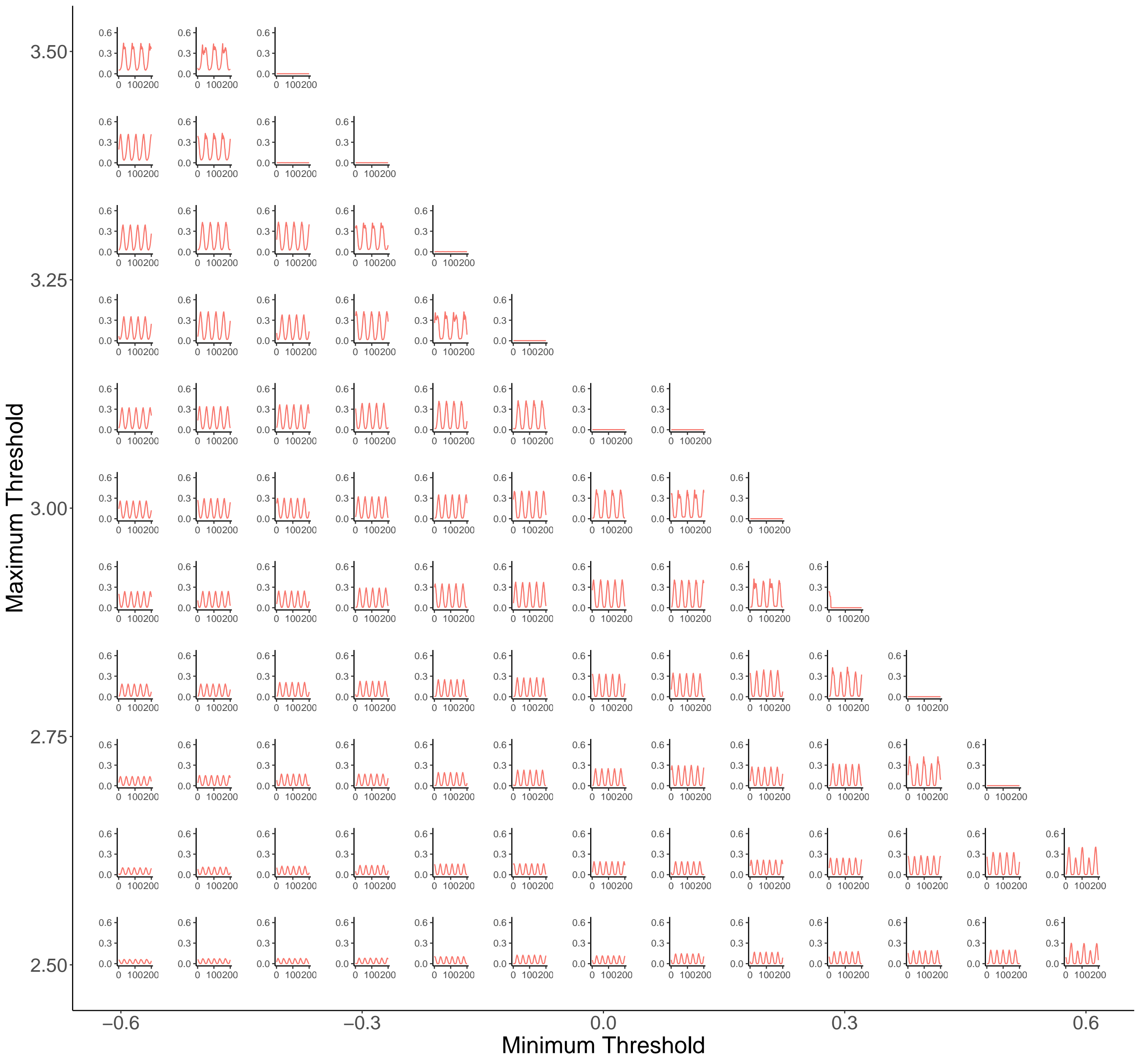

Supplement: S7 Fig — The threshold bounds for each simulation are [x, y], rounded to the nearest 0.1. For each sub-plot, the x-axis is the time (200 ticks) and the y-axis the fraction of signaling cells. Plots in the upper right with no signaling are cases in which the biofilm collapsed and extracellular potassium became extremely high. These data were used to create the phase diagram in Fig 6. (PDF) [file pcbi.1013746.s008.pdf]

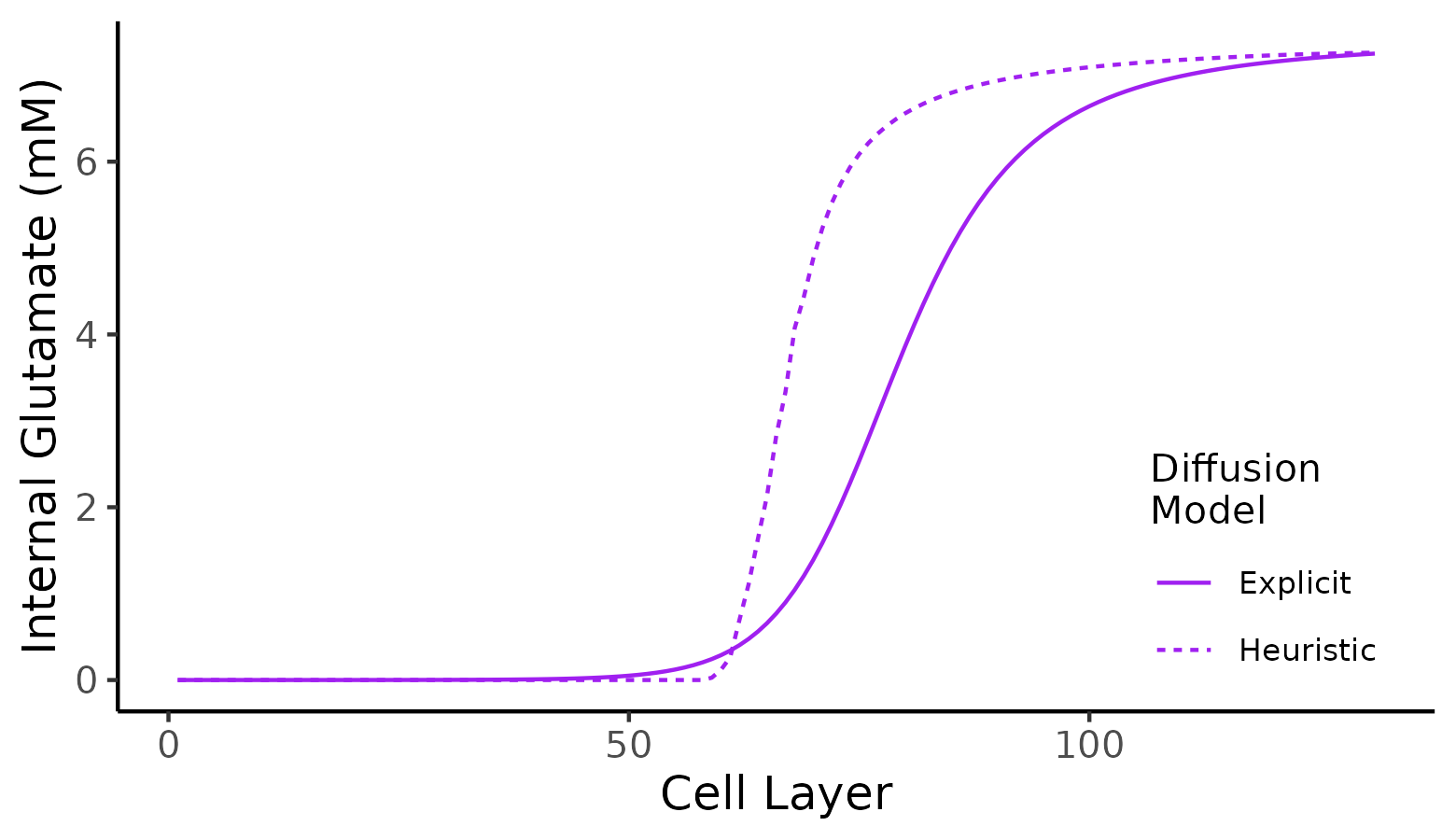

Supplement: S8 Fig — We removed cell signaling but retained glutamate uptake and degradation; the y-axis indicates mean internal glutamate in cells (results from a single simulation, which is effectively deterministic with these modifications). The x-axis is the distance from the center of the biofilm (in units of cells), with 0 being the center and a border at approximately 130 cells. The heuristic model shows a more abrupt drop-off in internal glutamate. (TIFF) [file pcbi.1013746.s009.tif]

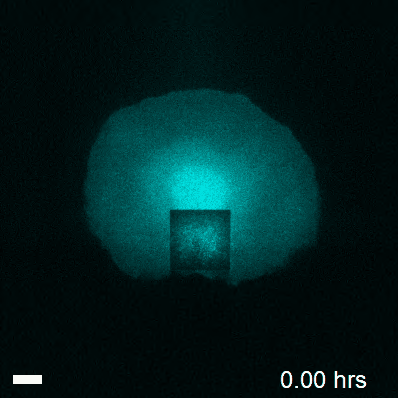

Supplement: S1 Video — This is a time-lapse of microscope images of a B. subtilis biofilm exhibiting the oscillatory behavior in vitro. The biofilm has been stained with the fluorescent membrane potential reporter ThT; cyan indicates hyperpolarized cells. The scale bar is 100 μm. (GIF) [file pcbi.1013746.s010.gif]
