# Supplementary material for: An agent-based model of metabolic signaling oscillations in Bacillus subtilis biofilms
Source: PLoS Comput Biol. 2025 Dec 4;21(12):e1013746. doi: 10.1371/journal.pcbi.1013746 (PMC12694845; doi:10.1371/journal.pcbi.1013746)
Supplement: S1 Table — Values for inner and total cells have been added in addition to outer cells. We also include consistency and inheritance estimates for the entire duration of signaling, not just pairwise, and with signaling measured across an entire oscillation, not only during the peak of signaling. Errors for all simulated results are standard deviations. For the signaling fraction and pairwise recurrences, standard deviations are estimated using 20 runs. For the rest, they are estimated using five runs. Error for the observed signaling fraction is a standard error from Zhai et al. [28]. Errors for observed signaling consistency are standard errors estimated as for a binomially distributed observation (number of cells = 316). (Standard error values were not reported in Zhai et al. [28]. We estimated them based on the given recurrence rates (0.6 and 0.78), fraction of signalers (43%), and number of observations (49 pairs of cells). This suggested 22 initial signalers, of whom 13 have signaling offspring, and 27 non-signalers, of whom 21 have non-signaling offspring. This produced the standard errors given above assuming binomially distributed counts.) (PDF) [file pcbi.1013746.s012.pdf]

	**Observed**	**Outer**	**Inner**	**Total**
Signaling Fraction	0.43 ± 0.02	0.42 ± 0.001	0.83 ± 0.048	0.43 ± 0.011
Pairwise Signaler Recurrence	0.60 ± 0.1†	0.63 ± 0.003	0.90 ± 0.029	0.68 ± 0.009
Pairwise Non-signaler Recurrence	0.78 ± 0.08†	0.73 ± 0.002	0.51 ± 0.027	0.74 ± 0.011
Total Signaler Recurrence	NA	0.48 ± 0.007	0.93 ± 0.003	0.71 ± 0.004
Total Non-signaler Recurrence	NA	0.72 ± 0.003	0.70 ± 0.009	0.69 ± 0.005
Pairwise Consistent Signaling Fraction	0.38 ± 0.03	0.41 ± 0.002	0.95 ± 0.003	0.44 ± 0.002
Pairwise Consistent Non-signaling Fraction	0.50 ± 0.03	0.57 ± 0.003	0.05 ± 0.000	0.55 ± 0.002
Pairwise Inconsistent Fraction	0.12 ± 0.02	0.02 ± 0.001	0.002 ± 0.000	0.01 ± 0.001
Total Consistent Signaling Fraction	NA	0.47 ± 0.001	0.75 ± 0.003	0.52 ± 0.001
Total Consistent Non-signaling Fraction	NA	0.52 ± 0.002	0.18 ± 0.001	0.46 ± 0.002
Total Inconsistent Fraction	NA	0.01 ± 0.000	0.07 ± 0.001	0.03 ± 0.001
