# Supplementary material for: An agent-based model of metabolic signaling oscillations in Bacillus subtilis biofilms
Source: PLoS Comput Biol. 2025 Dec 4;21(12):e1013746. doi: 10.1371/journal.pcbi.1013746 (PMC12694845; doi:10.1371/journal.pcbi.1013746)
Supplement: S2 Table — Content, formatting, and descriptions closely follow those in Martinez-Corral et al. [29]. The parameter that was labeled Dp in their paper has been changed to αk. Parameters divided by 50 were originally in units of hours but have been converted to be in units of ticks (t). (PDF) [file pcbi.1013746.s013.pdf]

Parameter	Description	Default Value	Units
t_{pp}	Tick size	50	hours^{-1}
gpt	Generation per tick	1/40	generations/tick
α_g	Glutamate uptake constant	24/50	mM/(μM t)
k_g	Extracellular glutamate concentration at half-maximal uptake rate	0.75	mM
δ_g	Glutamate degradation constant	5.7/50	mM^{-1} t^{-1}
α_k	Potassium uptake constant	0.282/50	mM^{-2} t^{-1}
K_{i0}	Homeostatic potassium setpoint	300	mM
F	Membrane capacitance	0.217	mM/mV
g_K	Potassium channel conductance	60/50	t^{-1}
V_{K0}	Nernst potential prefactor	25.8	mV
g_L	Leak conductance	18/50	t^{-1}
V_{L0}	Basal leak potential	-93.5	mV
V_{0T}	ThT fluorescence threshold	-95	mV
α_T	Maximal rate of ThT uptake	20/50	μM t^{-1}
γ_T	Intracellular ThT decay constant	10/50	mV t^{-1}
g_T	Inverse sensitivity of ThT to membrane potential	0.3	mV^{-1}
d_L	Leak slope coefficient	7.6	mV/mM
σ	Leak threshold sharpness coefficient	0.1	mV
V_0	Resting membrane potential	-86	mV
K_m	Basal potassium in media	8	mM
G_m	Basal glutamate in media	30	mM
$l_{\mathcal{T}}$	Lower threshold bound	0	mM
$u_{\mathcal{T}}$	Upper threshold bound	3	mM
$\sigma_{\mathcal{T}}$	Threshold standard deviation	1	mM
